# Small RNA Sequencing Uncovers New miRNAs and moRNAs Differentially Expressed in Normal and Primary Myelofibrosis CD34+ Cells

**DOI:** 10.1371/journal.pone.0140445

**Published:** 2015-10-15

**Authors:** Paola Guglielmelli, Andrea Bisognin, Claudia Saccoman, Carmela Mannarelli, Alessandro Coppe, Alessandro M. Vannucchi, Stefania Bortoluzzi

**Affiliations:** 1 Department of Experimental and Clinical Medicine, University of Florence, Florence, Italy; 2 Department of Molecular Medicine, University of Padova, Padova, Italy; 3 Department of Biology, University of Padova, Padova, Italy; IRCCS-Policlinico San Donato, ITALY

## Abstract

Myeloproliferative neoplasms (MPN) are chronic myeloid cancers thought to arise at the level of CD34+ hematopoietic stem/progenitor cells. They include essential thrombocythemia (ET), polycythemia vera (PV) and primary myelofibrosis (PMF). All can progress to acute leukemia, but PMF carries the worst prognosis. Increasing evidences indicate that deregulation of microRNAs (miRNAs) might plays an important role in hematologic malignancies, including MPN. To attain deeper knowledge of short RNAs (sRNAs) expression pattern in CD34+ cells and of their possible role in mediating post-transcriptional regulation in PMF, we sequenced with Illumina HiSeq2000 technology CD34+ cells from healthy subjects and PMF patients. We detected the expression of 784 known miRNAs, with a prevalence of miRNA up-regulation in PMF samples, and discovered 34 new miRNAs and 99 new miRNA-offset RNAs (moRNAs), in CD34+ cells. Thirty-seven small RNAs were differentially expressed in PMF patients compared with healthy subjects, according to microRNA sequencing data. Five miRNAs (miR-10b-5p, miR-19b-3p, miR-29a-3p, miR-379-5p, and miR-543) were deregulated also in PMF granulocytes. Moreover, 3’-moR-128-2 resulted consistently downregulated in PMF according to RNA-seq and qRT-PCR data both in CD34+ cells and granulocytes. Target predictions of these validated small RNAs de-regulated in PMF and functional enrichment analyses highlighted many interesting pathways involved in tumor development and progression, such as signaling by FGFR and DAP12 and Oncogene Induced Senescence. As a whole, data obtained in this study deepened the knowledge of miRNAs and moRNAs altered expression in PMF CD34+ cells and allowed to identify and validate a specific small RNA profile that distinguishes PMF granulocytes from those of normal subjects. We thus provided new information regarding the possible role of miRNAs and, specifically, of new moRNAs in this disease.

## Background

Philadelphia-negative chronic myeloproliferative neoplasms (MPNs) are a heterogeneous group of clonal hematopoietic stem cell (HSC) disorders associated with overproduction of mature myeloid cells[1,2].

MPNs are a group of chronic myeloid cancers that include essential thrombocythemia (ET), polycythemia vera (PV) and primary myelofibrosis (PMF), associated with an increased risk of thrombosis and/or hemorrhage and a propensity to develop acute myeloid leukemia.

Myelofibrosis, the most symptomatic and associated with the worst survival, can arise primarily or as a progression of PV or ET (post-PV/ET MF). In primary myelofibrosis (PMF) the increased proliferation of megakaryocytes is accompanied by deposition of fibrosis in the bone marrow, abnormal stem cell trafficking, and extramedullary hematopoiesis (myeloid metaplasia).[1,2]

In 2005, a new somatic mutation in the Janus Kinase 2 (JAK2)[3–7] has been discovered, providing the first genetic insight into the MPN pathogenesis. The JAK2V617F mutation is present in approximately 95% of patients with PV, and in 50% to 60% of those with ET or PMF. Subsequently, other mutations have been discovered, including mutation in MPL gene or in epigenetic regulators.[2,8–10]

In 2013, somatic mutations of *CALR*, the gene encoding calreticulin, have been found in 20% to 25% of patients with essential thrombocythemia (ET) or PMF[11,12]. Like *JAK2* and *MPL* mutations, somatic mutations of *CALR* behave as driver mutations responsible for the myeloproliferative phenotype.

Despite the fact that the mutational background of MPNs has been extensively investigated, the molecular etiology of the disease has not been fully elucidated. Indeed several lines of evidence indicate that the identified mutations are not sufficient for disease initiation and progression. Although murine models have provided unequivocal evidence that *JAK2* ^V617F^ is able to cause MPNs[13], disease phenotype is significantly heterogeneous between different murine lines and even within the same line, suggesting that disease phenotype is affected by other unknown genetic or epigenetic factors[14].

MicroRNAs are endogenous small non-coding RNAs, approximately 22 nt in length, crucial for post-transcriptional gene regulation. They are loaded into the RNA-induced silencing complex (RISC), directing the complex (including Argonaute proteins) to downregulate target mRNA expression by either triggering mRNA degradation or translational repression.[15] Recent studies show that mRNA destabilization explains most (66%–>90%) miRNA-mediated repression[16].

It is known that deregulation of miRNAs plays an important role in both solid and hematologic malignancies[17,18]. Indeed, hematopoietic differentiation is tightly governed by gene expression that is strictly regulated at multiple cell-fate decision levels. miRNAs regulate hematopoiesis acting both in HSC and in committed progenitor cells[18–20]. At the stem cell level, some miRNAs evolutionally conserved are responsible for expanding HSCs by inhibiting apoptosis[21–23]. At the progenitor cell level, miRNAs regulate the developmental fate of the megakaryocyte-erythroid progenitor (MEP) cell, the common progenitor of the erythroid and megakaryocytic lineages[24,25]. At the more committed hematopoietic cell level, specific miRNAs are expressed in different blood cell lineages and in different stages of hematopoietic differentiation. For example Chen et al.[26] reported that miR-142s expression was lower in the erythroid and T-lymphoid lineages and higher in B-lymphoid and myeloid lineages, while miR-223 expression was confined to myeloid lineages, with a very low detectable expression in T- and B-lymphoid and erythroid lineages.

miRNAs have an important role in regulation of hematopoiesis[27–30]. miR-16, miR-451 upregulation and miR-150, miR-155, miR-221 and miR-222 downregulation are associated with different stages of erythropoiesis[31,32]. miR-223 expression level, determined by two regulatory regions on its gene, fine-tunes lineage commitment of myeloid precursor[33]. miR-181 family was detected during granulocytic and macrophage-like differentiation and its level decreases along the hematopoietic lineage. It modulates differentiation by targeting and negatively regulating PRKCD mRNA, an upstream regulator of a pathway of the myeloid differentiation, and CAMKK1 mRNA, involved in the granulocytic and PMA-induced macrophage-like differentiation[34,35].

Recent studies highlighted aberrant miRNA expression in MPNs, and specific miRNA signatures that distinguish MPN granulocytes from those of healthy donors[18,36]. We also previously demonstrated that abnormal expression of miR-16-2 in vitro and in vivo contributes to the expansion of erythroid lineage in polycythemia vera.[20]

High-throughput array-based analysis of miRNA expression levels in MPN CD34^+^ cells were previously reported only by Lin et al.[35,37] A recent study characterized both gene and microRNA (miRNA) expression profiles in CD34+ cells from PMF patients[38]. It identified several biomarkers and putative molecular targets such as FGR, LCN2, and OLFM4. By means of miRNA-gene expression integrative analysis, the study suggested that JARID2 downregulation, mediated by miR-155-5p overexpression, might contribute to MK hyperplasia in PMF.

In a preliminary study, we performed short RNA massive sequencing and extensive bioinformatic analysis in the JAK2V617F-mutated SET2 cell line[39], detected and quantified 652 known mature miRNAs, of which 21 were highly expressed, thus being responsible of most of miRNA-mediated gene repression. In the same study, we showed that the majority of miRNAs were mixtures of sequence variants (isomiRs) and we identified 78 novel miRNAs. Indeed, we discovered that SET2 cells express a number of miRNA-offset RNAs (moRNAs), short RNAs derived from genomic regions flanking mature miRNAs, whose biological role needs to be elucidated.

In the present study, we characterized miRNA and moRNA expression in CD34+ stem cells using massive small RNA-seq. The observed specificities in small RNAs expression of PMF CD34+ cells were subsequently confirmed considering granulocytes from PMF, PV and ET patients and from healthy controls. We thus provided new information regarding the possible role of miRNAs and, specifically, of new moRNAs in the disease.

## Materials and Methods

### Cell-sample preparation and RNA extraction

CD34+ cells were purified from 30 to 50 mL PB collected from PMF patients or from 5 mL BM aspirate obtained in preservative-free heparin of healthy donors. All subjects provided informed written consent. The study, conducted in accordance with the Declaration of Helsinki, was approved by the Ethics Committee of the Azienda Ospedaliero-Universitaria Careggi of Firenze (Largo Brambilla 3, 50134 Florence, Italy), the 04/22/2011 (protocol n. 2011/0014777–37/11) in relation to the protocol "AGIMM (AIRC-Gruppo Italiano Malattie Mieloproliferative) Research Project".

Density gradient–separated mononuclear cells were processed through two sequential steps of immunomagnetic CD34+ selection (Miltenyi Biotec, Bergisch Gladbach, Germany, http://miltenyibiotec.com); final purity was evaluated by flow cytometry after labeling with PE-HPCA2 anti-CD34 monoclonal antibody (BD Biosciences, San Jose, CA, USA, http://www.bdbiosciences.com), and found to be >97% in all instances.

Total RNA was extracted using Trizol. Disposable RNA chips (Agilent RNA 6000 Nano LabChip kit; Agilent Technologies, Waldbrunn, Germany, http://www.home.agilent.com) were used to determine concentration and purity/integrity of RNA with Agilent 2100 Bioanalyzer.

Granulocytes were separated by differential centrifugation over a Ficoll-Paque gradient, starting from 20 mL PB; contaminating red cells were removed by hypotonic lysis, and cell pellets were resuspended in Trizol (Invitrogen Ltd, Paisley, UK, http://www.invitrogen.com).

### Small RNA-seq library construction and sequencing

For each sample, a small RNA library was prepared starting from 1 μg total RNA, using the TruSeq Small RNA Sample Preparation Kits and protocols (Illumina, San Diego, CA, USA, http://www.illumina.com). Library quality was checked using High Sensitivity DNA chip (Agilent Technologies, Waldbrunn, Germany, http://www.home.agilent.com). The purified cDNA libraries was used for cluster generation on Illumina's Cluster Station and sequenced on an Illumina HiSeq2000 instrument, producing single reads from 49 to 57 bp.

### Small RNA data analysis: preprocessing

After the adapter removal preprocessing step, reads “adapter-only”, too short or unclipped have been discarded. Unclipped reads are discarded because they can't represent miRNA or miRNA like short RNAs.

We admitted a read length range between 15 and 30 nt, slightly wider than the human annotated miRNAs length in miRBase to conserve also possible new longer isomiRs. We therefore discarded raw reads out of the range 15–30 bps in length. We then filtered out low quality reads, keeping all those reads displaying a mean base quality higher than 30, and allowing no more than 2 nucleotides per read with quality under 20. To complete data preprocessing we eliminated ground noise, considered as reads belonging to unique sequences with less than 10 reads counts each.

### Small RNA data analysis: reads mapping and comparative filtering

Reads have been mapped using Bowtie v. 1.1.0 both to the GRCh38 genome assembly and the known hairpins sequences extended in both directions by additional 30 bp to accommodate moRNAs mapping at the extremities of known hairpins. Reads mapping to more than 5 different loci on the genome, out of miRNA hairpins, are unlikely to be real miRNAs, and they have been thus discarded.

Barplot on Fig A in S1 File shows filtering effects on absolute reads counts for each sample.

We processed each sample data with our in-house pipeline miR&moRe. The output consists of lists of known miRNA read counts, lists of new miRNAs and moRNAs and lists of variants (isomiRs) for all the small RNAs found in each sample.

### Expression data normalization and sample cluster analysis

Sample merging and carefully conducted steps of data normalization and transformation are needed to guarantee the comparability of samples, to allow descriptive unsupervised analyses and differential expression tests. We performed normalization using R/Bioconductor package DESeq. Inference of differential expression in DESeq relies on the estimation of the typical relationship between the data variance and their mean, or, equivalently, between the data dispersion and their mean. Variance dependence to the mean can be modeled following different ways using DESeq, with several algorithms and very different results. The selection of the method used is crucial, since variance estimation influences unsupervised classification, differential expression and all following analyses. We tried two different methods for fitting data variance: 1) a parametric model, 2) a local regression model.

The first is the recommended default but in some data sets could fail to give optimal results. Sum of square of residuals for local regression is 15311.52 whereas for GLM is 156849.3, so we can conclude that local regression fits better our data.

We performed cluster analysis using R to check whether samples were correctly classified in their own biological class. We clustered samples using both full sRNAs expression matrix (917 sRNAs) and filtered, computing Euclidean distance and complete linkage as clustering method.

To filter the expression matrix at different levels of sRNAs expression we calculated, for each sRNA in the matrix, the sum of expression vector values. Then we performed clustering analysis under the following conditions 1) considering all the sRNAs found 2) selecting only sRNAs over the median (474 miRNAs and 26 moRNAs), 3) filtering only sRNAs over the third quartile (237 miRNAs and 12 moRNAs).

### Differentially expressed sRNAs

We performed a differential expression analysis using DESeq R/Bioconductor package. We considered those short RNAs that had a total expression throughout all samples higher than the median. We performed a multiple test correction according to the Benjamini Hochberg method (FDR). We considered a corrected p-value of 0.05 as threshold to identify differentially expressed elements.

### Validation of differentially expressed sRNAs

We performed individual miRNAs assay by Taqman quantitative real-time PCR (QRT-PCR) for quantification of abnormally expressed miRNAs in PMF and control granulocytes and in CD34+ cells. cDNA was synthesized from total RNA using microRNA-specific RT primers contained in the TaqMan microRNA Human Assays (Applied Biosystems). Briefly, single-stranded cDNA was synthesized from 10 ng total RNA in 15-μL reaction volume with the High-Capacity cDNA Archive Kit (Applied Biosystems) using 1 mM deoxyribonucleoside triphosphates, 50 U Multiscribe reverse transcriptase, 3.8 U RNase Inhibitor, and 50 nM of miR-specific RT primers. The reaction was incubated at 16°C for 30 minutes followed by 30 minutes at 42°C, and inactivation at 85°C for 5 minutes. Each generated cDNA was amplified by QRT-PCR with sequence-specific primers from the TaqMan microRNA Assays on an ABI Prism 7300 real-time PCR system (Applied Biosystems). PCR reactions included 10 μL 2× Universal PCR Master Mix (No AmpErase UNG), 2 μL each 10× TaqMan MicroRNA Assay Mix and 1.5 μL reverse-transcribed product; they were incubated in a 96-well plate at 95°C for 10 minutes, followed by 40 cycles of 95°C for 15 seconds and 60°C for 1 minute. Expression variations were calculated using the RQ method and a t-test p-value of 0.05 was used as threshold to identify differentially expressed elements.

### Target prediction of validated sRNAs and functional enrichment

The complexity of miRNA-mRNA interactions causes ambiguity in target prediction results. Target genes identification is indeed challenging and many algorithms have been developed. Target prediction programs can be divided in two classes, distinguished on the basis of the use or not of the information about evolutionary conservation of interaction[40]. We chose to perform a target prediction using two different programs, miRanda[41] and PITA[42], which implement orthogonal target prediction strategies. Our choice was determined also by code availability, which allowed us to make custom predictions using as query sequences also isomiRs and moRNA sequences.

We performed a target prediction using both miRanda 3.3a and PITA executable version 6 (31-Aug-08). We applied default parameters of miRanda to predict target of selected miRNAs and moRNA sequences since these settings are reported to optimize the dynamic programming miRanda algorithm. We used default parameters for PITA target prediction too. According to PITA documentation, we considered a binding site with score ≤ -10 likely to be functional in endogenous microRNA expression levels. We performed a hypergeometric test using an in-house modified version of the R Category package of Bioconductor, which supports Reactome annotation maps via the reactome.db R package.

### qRT-PCR analysis of target gene expression

For the analysis of expression level of *MECOM*, *MEIS1*, *AGO1*, *CAV1*, *AKR1C1*, *TIMP3*, *AGO3*, *FSTL1*, *JAKMIP2*, *TNSF10*, *BRCA1*, *PTPN4*, *MME*, *TCF4*, *FKBP10*, *SYS1*, *TRPS1* and RAN in CD34+ cells, RNA was reverse-transcribed with random hexamers and Murine Leukemia Virus (MuLV) reverse transcriptase (Thermo Fisher Scientific). qRT-PCR was carried out with TaqMan Universal PCR master mix, using TaqMan gene expression assays (*MECOM*: Hs00602795_m1, *MEIS1*: Hs01017441_m1, *AGO1*: Hs01084653_m1, *CAV1*: Hs00971716_m1, *AKR1C1*: Hs04230636_sH, *TIMP3*: Hs00165949_m1, *AGO3*: Hs01087121_m1, *FSTL1*: Hs00907496_m1, *JAKMIP2*: Hs00207662_m1, *TNSF10*: Hs00921974_m1, *BRCA1*: Hs01556193_m1, *PTPN4*: Hs00267762_m1, *MME*: Hs00153510_m1, *TCF4*: Hs00162613_m1, *FKBP10*: Hs0022557_m1, *SYS1*: Hs01110991_m1, *TRPS1*: Hs00936363_m1 and RAN: Hs03044733_g1; Thermo Fisher Scientific) by means of StepOne real-time PCR system (Applied Biosystems). Assays were performed in quadruplicate. Gene expression profiling was achieved using the RQ method as above, using RNase-P as the housekeeping gene.

## Results and Discussion

### Small RNA expression in CD34+ cells of patients with PMF

We considered small RNA sequencing data of 6 CD34+ cells, including 3 samples collected from 3 pools of bone marrow CD34+ cells of healthy subjects (CTR), and 3 samples of circulating CD34+ cells of patients affected by primary myelofibrosis (PMF), two of which were from individual patients while the third constituted a pool obtained by mixing equal amounts of RNA from 4 PMF patients. Samples and raw reads information are summarized in Table A in S1 File. The Illumina 2000 sequencing produced a total of 787,913,722 raw reads (131,318,954 per sample on average) that were deposited in Gene Expression Omnibus (GEO Series GSE69089).

After a stringent filtering during the preprocessing and quality control steps, 349,372,534 were aligned to GRCh38 genome “extended” hairpins (Fig A in S1 File). Aligned reads corresponded to 44.3% of initial raw reads, but due to high sequencing depth, the number of considered reads was still high.

We detected a total of 917 sRNAs, including 784 know miRNAs, expressed in at least one of the 6 considered CD34+ samples. Notably, 8 known miRNAs (miR-10a-5p, miR-181a-5p, miR-191-5p, miR-92a-3p, let-7a-5p, miR-146b-5p, miR-26a-5p and let-7f-5p) are highly expressed and contribute to the total miRNA expression throughout all samples from 2.5% to 25%, representing all together the 80% of the total expression. Moreover, we detected 133 new sRNAs, including 34 new miRNAs produced from known hairpins, and 99 microRNA-offset RNAs (moRNAs). Table 1 reports a summary of different types of sRNAs detected in the considered samples, according to current miRNA annotations.

**Table 1:** Summary of small RNAs expressed in CD34+ cells.

sRNAs	CTR	PMF	Total
known miRNAs	568	760	784
new miRNAs	20	31	34
moRNAs	52	96	99
Total new	72	127	133
Total	640	887	917

Cluster analysis and heatmaps of pairwise sample correlations are represented in Fig B in S1 File. Two heatmap plots were generated, one by considering normalized expression profiles of all the sRNAs expressed and another considering only those expressed over the median level. Both unsupervised analyses indicate that samples are correctly clustered according to sRNAs expression, with CTR samples clustered together and separately from PMF samples. Cluster analyses and heatmaps show that patients and controls sRNAs profiles are significantly different from each other and highlight a characteristic miRNA and moRNA expression profile in PMF.

### New miRNAs in CD34+ cells of PMF

In addition to 784 miRNAs annotated in miRBase, our in-house pipeline miR&moRe let us discover 34 new miRNAs expressed in considered samples (Table B in S1 File).

To find new miRNAs, we considered all the hairpins precursors annotated in miRBase, to be used as reference for read mapping and small RNA detection and quantification. Some of the listed hairpin precursors were known to only generate one mature miRNA with only a handful of reads reported in miRBase across all NGS experiments surveyed. After identifying the hairpin region that would most likely pair with annotated mature we classified as new miRNAs all the clusters of reads that map there. A consistent number of reads were attributed to new miRNAs. Since these reads passed stringent quality filtering and mapping criteria, it is improbable that they originate from sequencing errors. Furthermore, two of these new miRNAs, miR-2110* and miR-548ag-2*, resulted highly expressed and showed a mean expression over the median of expression values calculated on all small RNAs.

### miRNAs are mixtures of isoforms contributing to miRNAs expression

Microarray technology, relying on sequence hybridization to appropriately designed annealing probes, can only detect annotated miRNA sequences. Sequencing-based technology reveals instead all the repertoire of expressed small RNAs, both the unknown and the annotated miRNAs, and is able to detect isomiRs, that contribute to miRNA expression and qualitative characteristics. Our and other previous NGS studies showed that miRNAs are mixtures of sequences, slightly different from the official mature miRNA, called isomiRs [39,43].

In our dataset, miRNA expression counts indeed consider for each miRNA a group of reads that not only perfectly match the annotated miRNA (“exact”), but also match the precursor with a 1–2 nt shorter or longer sequence than the mature miRNA in the 3’ region (“shorter or longer at 3’”), in the 5’ end (“shorter or longer at 5’”) or at both the ends (“both”).

Expressed miRNAs with unique sequence are very few, only 165 out of the 784 annotated miRNAs detected (21%). Unique sequence miRNAs are, in general, weakly expressed: they were detected at level under the median of the mean expression values distribution. Notably, the remaining 619 expressed miRNAs have more than one isomiR. We also detected reads aligning to hairpin precursors with one or two mismatches but we excluded these reads from the total miRNAs expression estimation.

**Fig 1:**
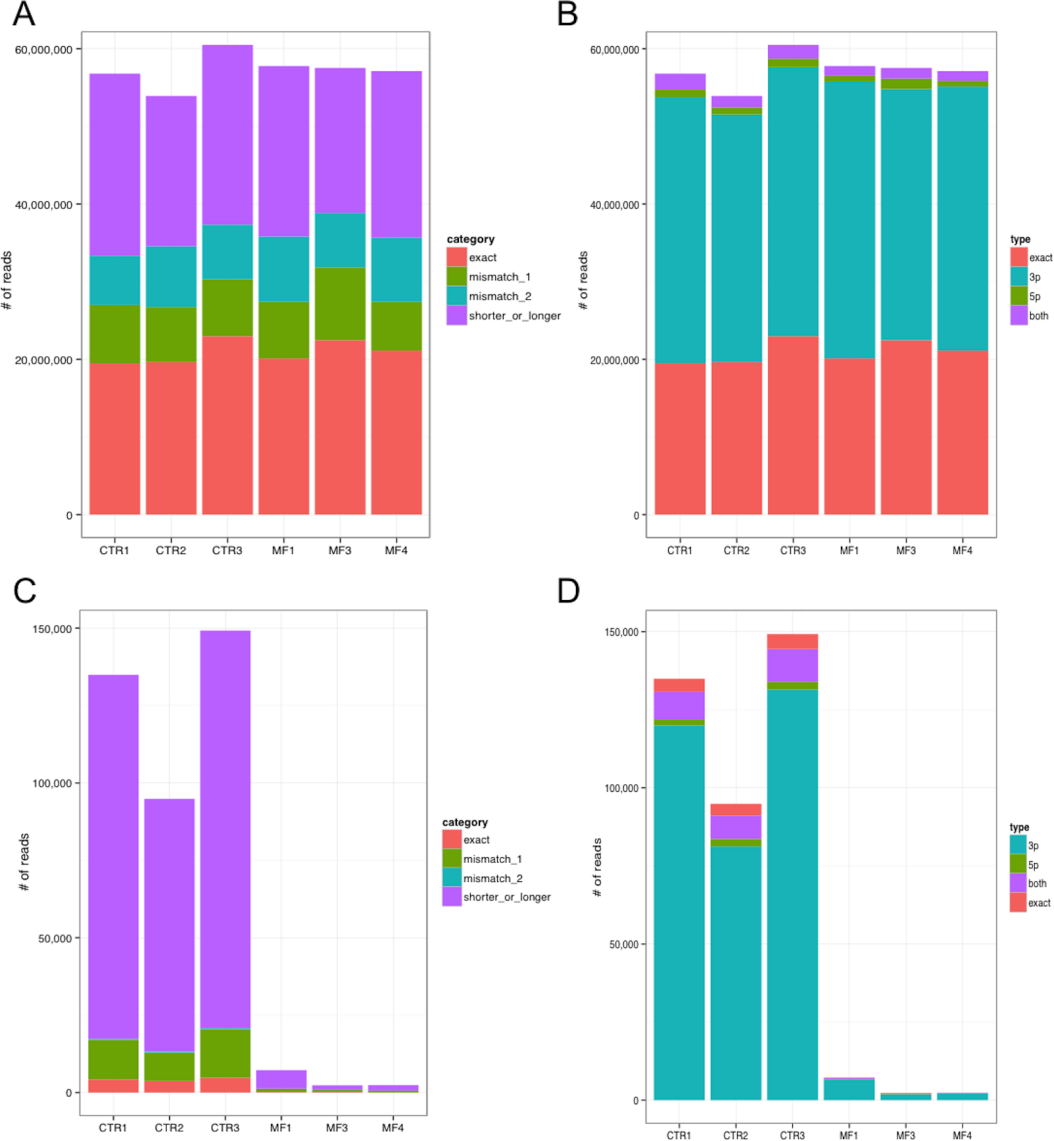
Distribution of reads per isomiR category (A) and per type (B) across samples, considering all 818 expressed miRNAs. “Exact” reads are identical to the mature miRNA sequence annotated in miRBase, whereas “mismatch” reads present respectively one or two nucleotides different from the annotated sequence but identical length; the last category includes reads perfecly matching the miRNA precursor (and genomic) sequence but shorter or longer than the annotated mature miRNA. IsomiR types indicate if the sequence difference fall in the 5’ region of the miRNA, in the 3’ region, or in both regions. Abundance of reads falling in different isomiR categories (C) and types (D) for miR-10b-5p, showing that isomiRs different from the annotated mature miRNA sequence are very abundant.

Fig 1A shows that the annotated sequence, called “exact” isomiR, contributes on average to 36.6% of the miRNA expression, whereas a considerable part of the total expression contribute is given by shorter or longer isomiR sequences (37.3% of expression, in average). Reads aligning with mismatches are also represented in display items for comparison (Fig 1A). We investigated if sequence differences observed in non “exact” isomiRs involve the 5’ half of the miRNA, including the seed region crucial for target recognition, the 3’ half, or both. Fig 1B shows that, considering all expressed miRNAs, most differences regard the 3’ half of the miRNAs, and isomiRs with 5’ region different from the “exact” sequence are very rare. Considering isomiR type and miRNA region involvement together, we observed that mismatch isomiRs impacting on the 5’ region were not detected at all (Fig C in S1 File). Considering the top 25% most expressed miRNAs, the picture is basically unchanged (Fig D-A-C in S1 File). Looking at specific miRNAs, the ratio between isomiR types changes. For example, miR-10b-5p is mainly detected in its “shorter or longer” variant (Fig 1C and 1D).

IsomiRs, initially discarded as sequencing/alignment artifacts or poor quality RNAs,[44–47] were lately demonstrated to be highly expressed with an elevated degree of confidence. They were experimentally validated and characterized as tissues, conditions and cell types specific[48–50]. Despite isomiR biogenesis being still unclear, tissue specificity and differential expression during different development stages suggest to be ruled by a fine-tuned mechanism. Several lines of evidence suggest that isomiRs could be processed by an alternative Drosha/Dicer cleavage of the pre-miRNA[51–53] or by editing/nucleotide addition of the mature annotated miRNA[54].

Regarding isomiR biological role, they were demonstrated to be biologically functional and to work similarly to the canonical miRNA. In fact, Azuma-Mukai et al. [48] and Chin Tan et al.[50] co-immunoprecipitated isomiRs with Ago proteins, supporting isomiRs biological activity. Cloonan et al.[55] biotin-labeled miRNAs and isomiRs, to pull down endogenous mRNA targets: after ultra-deep miRNA-seq in different tissues, isomiRs expression levels resulted comparable to the canonical miRNAs. Reportedly, isomiRs are incorporated into RISC complexes binding endogenous mRNAs. The fact that 5’ isomiRs are also under selection during evolution witnesses their functional importance[50].

Anyway, sequence differences, and notably those in the seed region can potentially direct different isomiRs to diverse target mRNAs. Indeed 5’isomiRs target different mRNAs than their canonical counterparts[50]. Chan et al.[56] showed that different miR-31 isomiRs are expressed in different cell types and, notably, that even sharing the same seed region, slightly different miR-31 isomiRs exert different degree of repression of verified target genes.

Considering that different isomiRs can characterize different tissues or conditions, as tumor respect to normal tissues, and that diverse isomiR could impact differently on target genes and pathways, isomiR deregulated expression could be implicated in disease. We thus considered the possibility that peculiar genetic characteristics of the considered PMF cells would result in specific isomiR sequences, or could be related to variations of isomiRs expression level in disease. We searched for mismatch isomiRs expressed in PMF samples and not in CTR samples, possibly due to genetic mutations: 44 isomiRs belonging to 30 expressed miRNAs were identified only in PMF samples, but they resulted weakly expressed, with a read count lower than 400, and/or not accounting for at least the 25% of the corresponding miRNA expression (data not shown).

We searched also for differentially expressed isomiRs comparing PMF and CTR samples. 133 isomiRs from 59 miRNAs were associated to a Log of Fold change at least 1 and adjusted t-test p-value lower than 0.05. Considering all isomiRs accounting each for at least 10% of mature miRNA expression, we observed that isomiRs and miRNA expression profiles were highly correlated (75% of pairwise correlation values over 0.699, median correlation of 0.917; Fig E-A in S1 File). Similarly, also differentially expressed isomiRs resulted highly correlated with miRNAs (75% of pairwise correlation values over 0.743, median correlation of 0.895; Fig E-B in S1 File).

Anyway, since miRNAs are *de facto* mixtures of isomiRs, specific variations of isomiRs expression impact also on miRNAs expression. Thus, we considered isomiR counts for miRNA expression calculations and for the following analyses aiming at investigating the impact of miRNAs and on moRNAs in specific functions and pathways.

### moRNAs discovery

As anticipated, we also detected in our samples sequences aligning to hairpins outside known and novel miRNAs, that correspond to expressed microRNA-offset RNAs, called moRNAs (Table 1). moRNAs sequences partially overlap miRNA regions but generally span the Drosha cutting sites, letting us hypothesize a non canonical processing of the hairpin precursor in moRNA biogenesis[39].

A complete list of all the detected moRNAs is in Table C in S1 File. Noteworthy, 28 moRNAs were highly expressed, 26 of them over the median of the short RNAs expression values distribution and 12 of them even over the third quartile (Table 2).

**Table 2:** List of most abundant moRNAs in considered CD34+ samples, which are expressed over the third quartile of all sRNAs expression. For each moRNA, the table reports expression (per sample group normalized read count), position and sequence.

moRNA	Average read count CTR	Average read count PMF	Strand	Position	Sequence
moR-128-2-3p	2493	0	+	chr3:35744548–35744568	CCCTACTGTGTCACACTCCTA
moR-21-5p	2946	2437	+	chr17:59841243–59841271	ACATCTCCATGGCTGTACCACCTTGTCGG
moR-24-2-5p	5971	1719	-	chr19:13836350–13836376	TGCCTGGCCTCCCTGGGCTCTGCCTCC
moR-27a-5p	1752	312	-	chr19:13836510–13836534	CGAAGCCTGTGCCTGGCCTGAGGAG
moR-3651-5p	0	1266	-	chr9:92292537–92292565	ATGGACAGCTCTCCAGTGGATTCGATGGG
moR-421-5p	848	2784	-	chrX:74218449–74218472	CCTAATCCGGTGCACATTGTAGGC
moR-6724-1-5p	683	280	+	chr21:8205298–8205332	TGTGGGGGAGAGGCTGTCGCTGCGCTTCTGGGCCC
moR-6724-2-5p	683	280	+	chr21:8249488–8249522	TGTGGGGGAGAGGCTGTCGCTGCGCTTCTGGGCCC
moR-6724-3-5p	683	280	+	chr21:8388345–8388379	TGTGGGGGAGAGGCTGTCGCTGCGCTTCTGGGCCC
moR-6724-4-5p	683	280	+	chr21:8432513–8432547	TGTGGGGGAGAGGCTGTCGCTGCGCTTCTGGGCCC
moR-941-4-5p	6564	2531	+	chr20:63919746–63919768	CACCCGGCTGTGTGCACATGTGC
moR-941-5-5p	9780	3800	+	chr20:63919858–63919880	CACCCGGCTGTGTGCACATGTGC

We classified moRNAs on the basis of the hairpin precursor arm they where processed from: 5’-moRNAs mapping to the 5’ hairpin arm, and 3’-moRNAs spanning over the 3’ hairpin arm. 5’-moRs were significantly more abundant respect to 3’-moRs. Out of 99 moRNAs expressed in considered samples, only 16 (16.2%) were processed from the 3’ hairpin arm, while 83 (83.8%) were 5’-moRs. According to our data, seven hairpins were processed producing two moRNAs each (Table C in S1 File).

5’-moRs estimated expression values were 10 times higher than 3’-moRs, ranging from summed up normalized values over all samples of 5 to 40,739, compared to a 3’-moRs range of 6 to 7,478. Both 3’-moRs and 5’-moRs are more expressed in controls than in PMF patient samples.

**Fig 2:**
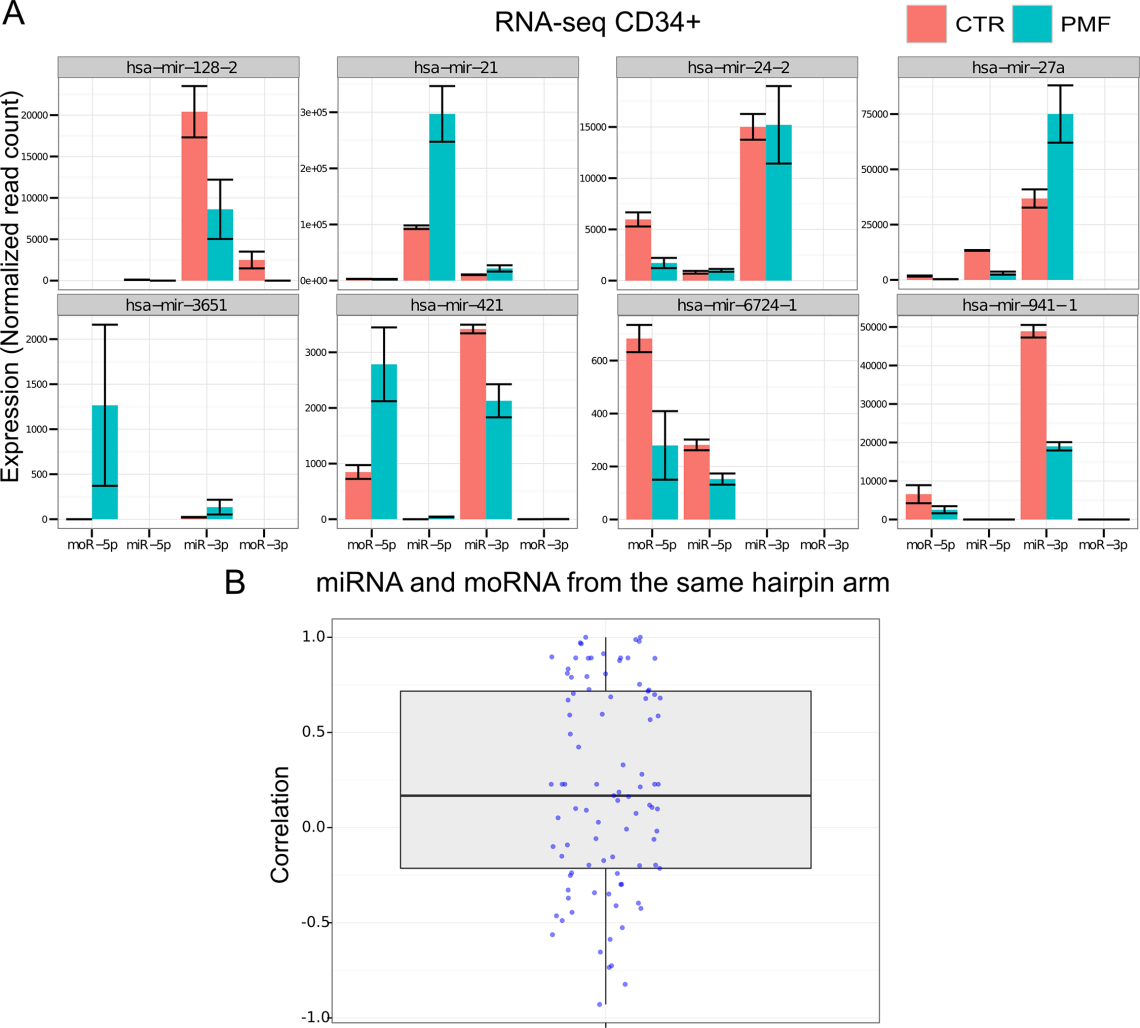
A) Expression, in CD34+ cells of PMF patients and controls, of miRNAs and moRNAs produced from the same hairpin, considering the hairpins expressing most abundant moRNAs; the boxplot in panel (B) shows the distribution of Pearson correlation values calculated pairwise between expression profiles of moRNAs and miRNAs produced from the same hairpin arm, considering all moRNAs detected in CD34+ cells.

Considering the 9 most expressed moRNAs, Fig 2A shows expression estimations in PMF and CTR samples of all the expressed small RNAs that are produced from the same hairpins precursor. Specific hairpins, as hsa-mir-421 and hsa-mir-941-1, reported in Fig 2A, express two moRNAs each.

Considering the miRNA and the moRNA produced from the same hairpin arm, we noted that in 4 out the 9 reported in Fig 2A the moRNA is more expressed than the miRNA and in one the miRNA close to the moRNA is not detected at all. Moreover, some hairpins show a similar trend toward up- or down-regulation for both the miRNA and the moRNA, whereas in others the two sRNAs show opposite behavior. We then compared the expression behavior of all expressed moRNAs with that of the miRNA coming from the same hairpin arm. The boxplot in Fig 2B shows the distribution of 93 Pearson correlation values calculated pairwise between expression profiles of detected moRNAs and miRNAs from the same hairpin arm: the values range from -0.93 to +1, with median and mean values of 0.17 and 0.21, indicating a very slight tendency toward positive correlation, the presence of abundant anticorrelated pairs (36, 38.7%). Considering the statistical significance of correlation, only 7 pairs (7.5%) resulted significantly correlated (with a q-value at most 0.1); since they include an anticorrelated pair (5’moR-93 and miR-93-5p) and do not overlap with the group of highly expressed moRNAs, we can conclude that moRNA expression is largely independent from that of the close miRNA.

Even if moRNAs mechanism of action is still unknown, their considerable high expression level in this dataset together with previously reported observations [39,43,57] offers an indirect but intriguing indication of a biological role.

### Identification of sRNA differentially expressed in PMF vs CTR

Since myeloproliferative disorders are clonal hematopoietic stem cell neoplasias, miRNA and moRNA deregulation can be implied in tumor physiopathology.

**Fig 3:**
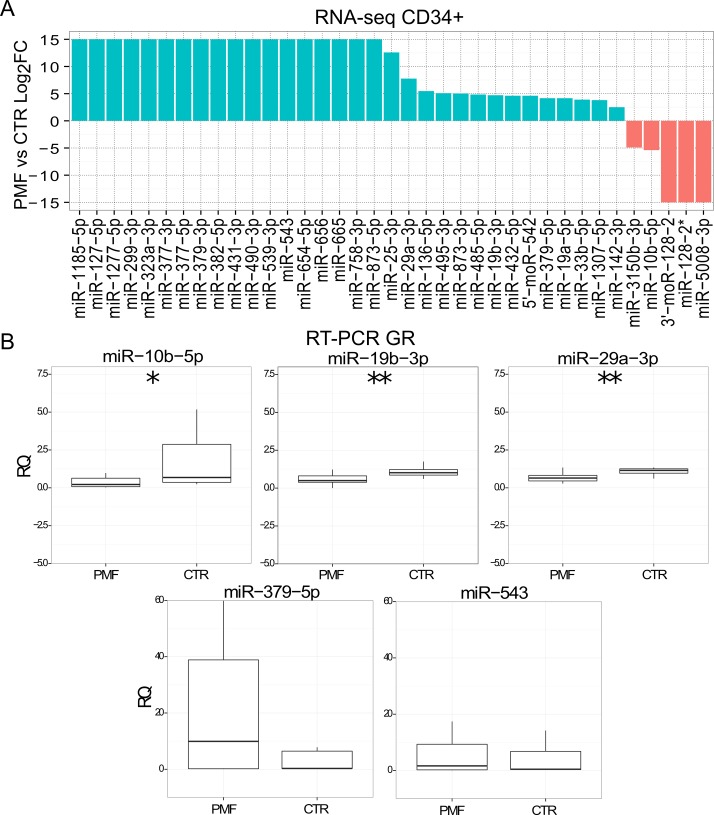
Differential expression of small RNAs in PMF vs CTR CD34+. A) Log2 FC of 37 small RNA differentially expressed considering PMF vs CTR CD34+, according to RNA-seq data (FDR<0.05). When a small RNA was not expressed in one sample category, the ratio was infinite and we represent it as the arbitrary maximum value of 15. B) RT-PCR expression calculation of five selected miRNAs in granulocytes collected from an independent cohort of normal controls (n = 10) and of PMF (50) samples; ***, ** and * indicate respectively a p-value <0.001, <0.01 or <0.05.

We recognized 37 sRNAs significantly differentially expressed (DE) in PMF patient samples respect to control CD34+ (Table D in S1 File). Fig 3A shows the logarithm of the mean expression ratio in PMF and control cells for DE miRNAs and moRNAs. While only five small RNAs are downregulated, the majority of DE sRNAs resulted upregulated in PMF patients.

Noteworthy, among the differentially expressed sRNAs, 2 moRNAs (5’-moR-542 and 3’-moR-128-2) are included. Incidentally, the two miRNAs (miR-542-5p and miR-128-3p) that are produced by the same hairpin arm of differentially expressed moRNAs are not differentially expressed in the same sample comparison. 5’-moR-542 is up-regulated in PMF with a log_2_FC of 3.5. 3’-moR-128-2 is highly expressed in normal CD34+ cells, at levels over the third quartile of the overall small RNA expression distribution, and dramatically downregulated in PMF patients: the moRNA was not detected in considered PMF samples. We mapped 3’-moR-128-2 sequence to the whole human genome to exclude multiple matching loci and to rule out mapping or annotations artifacts. We can thus exclude that moRNA-associated reads could come from different or contaminating RNAs. An additional UCSC Blat[58] analysis confirmed that the moRNA sequence only aligned to a single genomic locus (chr3:35786042–35786062). We are therefore confident that the detected small RNA is a moRNA derived from the non-canonical processing of the human mir-128-2 hairpin.

### Validations of selected differentially expressed miRNAs in PMF granulocytes

**Fig 4:**
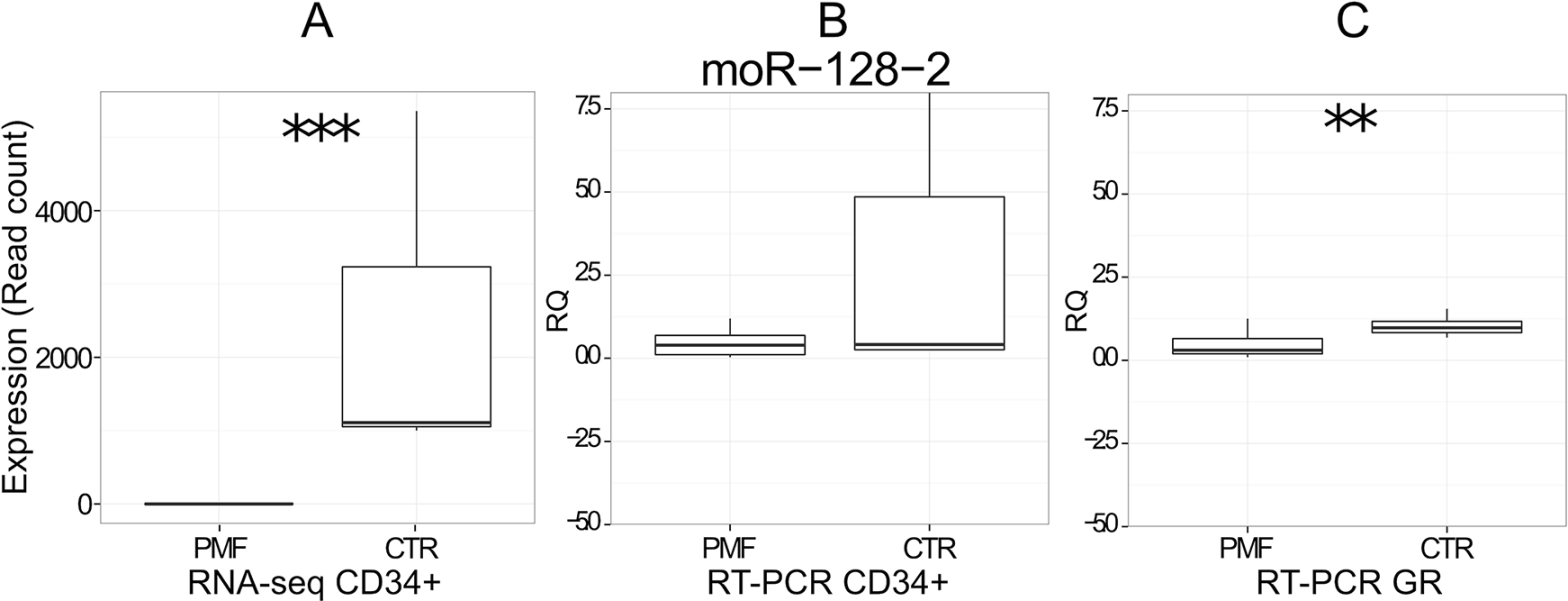
Differential expression of 3’-moR-128-2 in PMF (n = 3) vs CTR (3) cells. A) moR expression in PMF and CTR CD34+ according to RNA-seq data. B) RT-PCR expression (RQ) in CD34+ cells from independent cohort of normal controls (n = 8) and of PMF (20) samples. C) RT-PCR expression (RQ) in granulocytes from independent cohort of normal controls (n = 10) and of PMF (50) samples; ***, ** and * indicate respectively a p-value <0.001, <0.01 or <0.05.

We selected the most significantly deregulated and highly expressed miRNAs for further analysis. Specifically, we considered 6 miRNAs among the differentially expressed for a quantification with Real-time PCR (RT-PCR) in granulocytes collected from an independent and sizeable cohort of normal controls (N = 10) and PMF (N = 50), PV (N = 30) or ET (N = 30) patients. miR-10b-5p and moR-128-2 resulted significantly downregulated in PMF granulocytes samples, as according to RNA-seq data in CD34+ cells (Figs 3B and 4B). Two additional miRNAs (miR-379-5p and miR-543) showed in PMF granulocytes the same trend toward upregulation in PMF granulocytes (Fig 3B) as in CD34+ cells by RNA-seq data. Both miR-29a-3p and miR-19b-3p resulted significantly downregulated in PMF granulocytes (Fig 3B).

Furthermore, among the six small RNAs considered for validation in PMF granulocytes, 5 showed the same trend of changes also in ET or PV granulocytes, whereas miR-543 resulted upregulated in PMF and ET, and downregulated in PV, with small variations (Fig F in S1 File). miR-379-5p was upregulated in ET and in PV, as in PMF; miR-543 was slightly increased in ET, as in PMF, but not in PV; miR-19b-3p and moR-128-2 showed a similar trend, being decreased both in ET and in PV, but at a lower extent than in PMF; miR-10b-5p was decreased in ET and in PV, also more than in PMF. Notably, considering the statistical significance of the observed variation, miR-10b-5p is significantly downregulated both in PV and ET samples, indicating that it is associated with MPN in general more than being specific for PMF.

Of interest, miR-10b-5p resulted downregulated both in PMF CD34+ and granulocytes. Accordingly, miR-10b-5p has been previously reported to be deregulated in breast cancer[59–61]and involved in chemoresistance related pathway[62]. It has been validated as downregulated in endometrial carcinoma[63], bladder cancer [64], in advanced stage of small cell carcinoma of the cervix (SCCC)[65] and in clear cell renal cell carcinoma (ccRCC) and its expression level has been also included in a linear model that capture the metastatic tumor signature and patient prognosis[66].

We found miR-29a-3p upregulated in patients CD34+ respect to controls, but downregulated in the same comparison when considering committed granulocytes. This observation is in accordance with results previously obtained by Han et al.[67] who showed that miR-29a-3p is expressed at lower levels in hematopoietic progenitors compared to lineage-committed progenitors, including granulocytes, overall indicating that its expression level increases along with commitment. Therefore, we point to miR-29a as a deregulated small RNA in PMF, whose expression is uniquely modulated along myeloid differentiation of PMF CD34+ cells. Consistently with our finding, Han et al. also showed also that sustained expression of miR-29a-3p in mouse HSC/progenitors pushed myeloid progenitors to self-renewal capacity, to biased myelopoiesis and to the onset of a myeloproliferative disorder that progressed to acute myeloid leukemia. Additional data supporting miR-29a-3p deregulation derives from a previous study by Norfo et al.[38], in which miRNA expression profiling was obtained by Affymetrix miRNA 2.0 array analysis in a cohort of 42 PMF patients CD34+ cells and 31 healthy donors. In that study, miR-29a-3p was found upregulated in CD34+ from PMF patients as well as, miR-29a-3p upregulation in PMF CD34+ cells was validated by RT-PCR (with TaqMan probes) in an independent set of CD34+ cells from 10 PMF patients and 8 healthy subjects.

In the study of Norfo et al., also the upregulation of miR-379-5p, miR-543 and miR-19b-3p were validated in PMF CD34+. In present study, we were unable to confirm a statistically significant upregulation of these miRNAs in PMF, although there was a trend toward for miR-379-5p and miR-543. Interestingly, Norfo et al. validated microarray-based observations using two sets of qRT-PCR experiments, conducted on CD34+ cells and on granulocytes, showing that PMF-specific variations of a few miRNAs may be observed in CD34+ cells unlike in granulocytes. Indeed, miR-486-3p was significantly downregulated in PMF granulocytes and upregulated in PMF in CD34+ cells.

In summary, available evidence indicate that miR-10b-5p and moR-128-2 are downregulated in PMF CD34+ cells, whereas miR-19b-3p, miR-379-5p and miR-543 are upregulated. For these miRNAs the evidence of differential expression in PMF was robust, since it was detected by NGS and also validated technically (by qRT-PCR) and biologically (in independent samples).

### 3'-moR-128-2

3’-moR-128-2, a newly annotated small RNA, resulted expressed in CD34+ while it was not detected in PMF. We evaluated its expression level with qRT-PCR both in CD34+ cells and in granulocytes. RT-PCR validation in CD34+ cells were conducted considering 8 normal controls and 20 PMF patients, whereas the validation in granulocytes was done with the same design and samples used for miRNA validation. Fig 4 shows the consistent downregulation of 3’-moR-128-2 in PMF patients according to RNA-seq (Fig 4A) and RT-PCR data in CD34+ (Fig 4B) and to RT-PCR data in granulocytes (Fig 4C)**.** Moreover, 3’-moR-128-2 was decreased, but at a lower extent, without reaching statistical significance, also in PV and ET granulocytes (data not shown).

Intrigued by the striking expression pattern of the newly discovered 3'-moR-128-2 we looked in details into sequence, structure, expression and functional differences of 3'-moR-128-2 and miR-128-3p.

**Fig 5:**
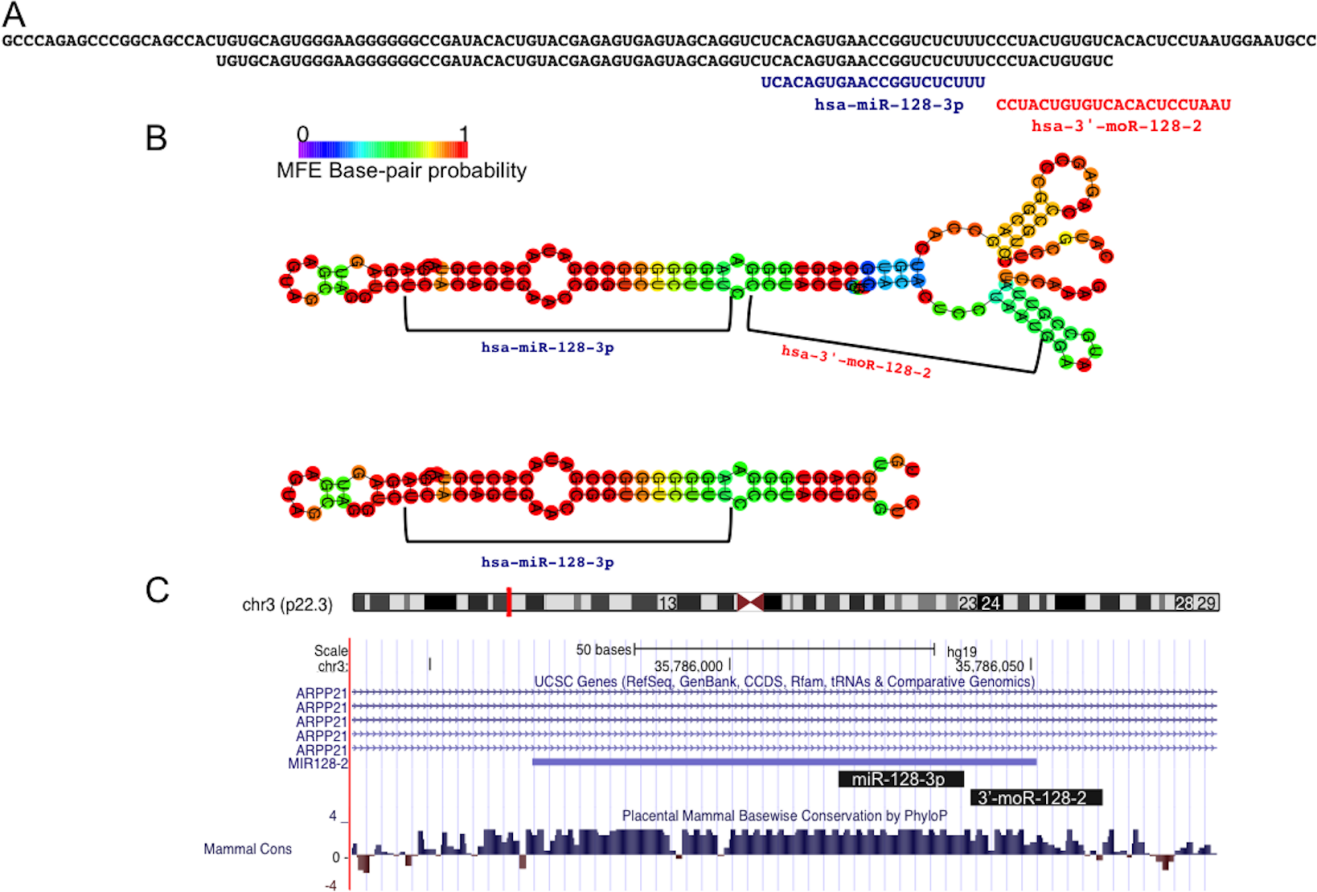
The 3’-moR-128-2 is produced by the precursor sequence of miR-128-3p. A) The moRNA is derived from a region of the primary miRNA sequence exceeding the canonical hairpin precursor sequence, and it is not exaclty adjactent to the annotated miRNA. B) Minimum free energy (MFE) folding structure, predicted by RNAfold, for the canonical hairpin sequence and for the longer one, from which the moRNA is probably derived. C) Both the considered small RNAs are conserved in evolution through vertebrates.

In Fig 5 we show additional sequence information regarding miR-128-3p and 3’-moR-128-2. Since the moRNA sequence is not contained in the canonical hairpin (Fig 5A), the moRNA probably derives from the processing of an alternative hairpin precursor. In Fig 5B we show the RNAfold predicted minimum free energy (MFE) folding structure of the canonical hairpin and of a longer sequence from which the moRNA can be derived. Fig 5C shows that mir-128 locus is inside an intron of ARPP21 gene, and displays the genomic region Mammals UCSC base-wise PhyloP conservation score.

**Fig 6:**
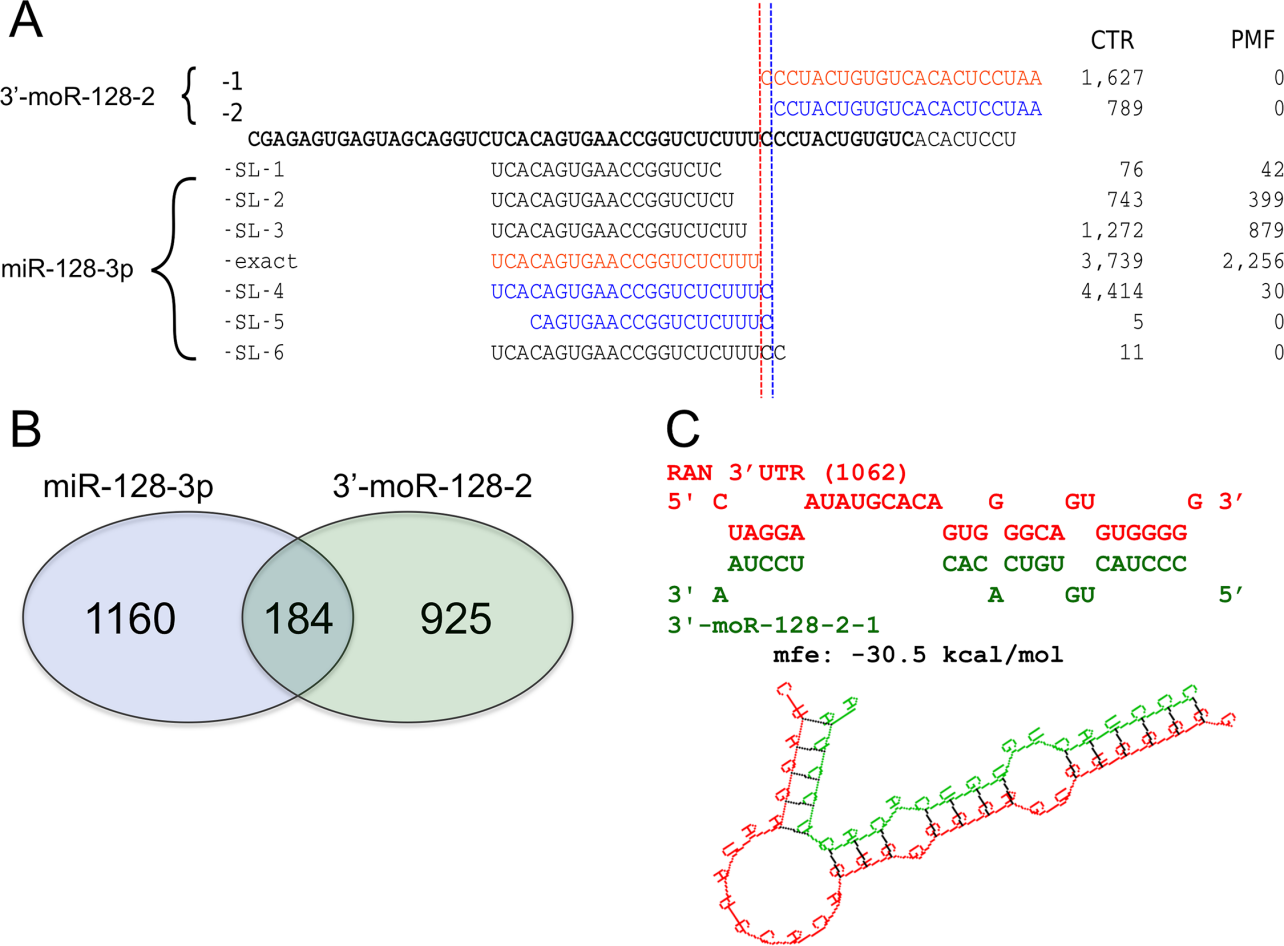
Origin, sequence variability, and relations beween 3’-moR-128-2 and the adjacent miR-128-3p. A) 3’-moR-128-2 and miR-128-3p map to the same locus and both shows sequence variability (isomiRs and isomoRs). Both the major and the minor isomoRs are found in normal CD34+ cells and not in PMF samples. Red and blue colors indicate isomiR and isomoR groups that can be produced with an unique sequence cutting sites. The most expressed isomoR is not associated to the corresponding most expressed isomiR. Moreover, expression levels, in CTR and PMF samples, of isomiRs and isomoRs are poorly correlated intragroup. These observations, point against the moRNA being simply a by-product of the miRNA biogenesis. A similar indication is given by the fact that some abundant isomiRs are not associated to detected isomoR sequences. B) 3’-moR-128-2 and miR-128-3p have different, poorly overlapping, sets of predicted targets. C) 3’-moR-128-2 sequence can stably bind a target site in the 3’UTR of the RAN mRNA, causing post transcriptional silencing.

moRNA biological roles and mechanisms of function still deserve investigation. Very likely, moRNAs can function as miRNAs in post-transcriptional gene silencing, guiding RISC to complementary target mRNAs. This was first demonstrated by Umbach and colleagues, that used a luciferase-based indicator assay to demonstrate that a viral moRNA (moR-rR1-3-5p) has inhibitory activity against an artificial mRNA bearing a perfect target site [68,69]. Beyond this proof of principle experiment, a recent study reported moRNA specific expression in human embryonic stem cells[70] (hESCs). In the same study, moRNA and miRNA transfection experiments and microarray quantification of gene expression were conducted and identified gene silenced by moR-103a-2-3p, one of the most abundantly expressed moRNAs in hESCs, and by miR-103a. In line with these previous studies, we assumed that 3’-moR-128-2 can act as a miRNA, and investigated its possible impact on target gene silencing and on specific pathways or biological processes. To this end, we considered how sequence variants (isomiRs) of these two small RNAs relate to each other (Fig 6). For miR-128-3p, we identified 7 variants expressed in considered CD34+ samples: one exact isomiR, corresponding to the miRBase annotated mature form, and 6 “shorter or longer” variants (miR-128-3p-SL-1 to miR-128-3p-SL-6), whereas only 2 3'-moR-128-2 isomoR were found out (3'-moR-128-2-1 and 3'-moR-128-2-2)(Fig 6A).

According to the conservative hypothesis that interprets moRNA as byproducts of Drosha cleavage[71] one should expect comparable mean levels of miRNA and moRNA cognate variants (i.e. obtained from a single endonucleolytic cleavage cut) in PMF and CTR samples. The observation that 3'-moR-128-2-1, the most abundant isomoR, is expressed only in CTR samples and its only viable cognate partner is the miR-128-3p-exact variant that is highly and nearly equally expressed in CTR and PMF samples (with 3,739 and 2,256 normalized reads, respectively)(Fig 6A) does not point in this direction. Neither do the poor correlation of expression levels, in CTR and PMF samples, of cognate isomiRs and isomoRs, the fact that some abundant isomiRs are not associated to detected isomoR sequences, the good conservation of moRNA sequence (Fig 5C), and the previously reported observation that both isomoRs are not contained in the canonical hairpin (Figs 5A and 5B and 6A).

### Genes and pathways targeted by the sRNAs deregulated in PMF

We conducted a preliminary functional characterization of the possible biological role of sRNAs DE in PMF, by a double strategy. First we investigated possible target genes and pathways of the group of validated DE sRNAs, considered as a whole. Then, we focused on one of the most novel elements emerged by our results, 3’-moR-128-2, to get specific insights on its possible functions in CD34+ and, in turn, in PMF disease.

Target predictions of miR-10b-5p, miR-19b-3p, miR-29a-3p, miR-379-5p, miR-543 and of moR-128-2 were performed by using two different programs, miRanda[41] and PITA[42], which implement orthogonal target prediction strategies, and for which the code availability allowed us to make custom predictions of possible miRNA and moRNA target genes, by using as query sequences the identified isomiR and isomoR sequences.

Among different isomiRs detected for each considered miRNA, we considered the most expressed, even if it was different from the annotated sequence (Table E in S1 File). We also considered those isomiRs that were significantly contributing to miRNA total expression, and which were differently expressed in patients respect to controls (t-test p-value <0.05 and |log_2_FC|>1), according to RNA-seq data. Accordingly, both isomoRs were considered for moR-128-2.

A functional enrichment analysis, based on Reactome annotation maps, of targets predicted by both methods was obtained using a hypergeometric test.

Table F in S1 File reports, the Reactome pathways enriched (q-value < = 0.05) among the union of predicted target genes of miRNAs and moRNAs differentially expressed in PMF vs CTR samples, giving some hints on the pathway more specifically targeted, collectively, by short RNAs deregulated in PMF. These enrichments are based on the subset (1,521; 36%) of 4,222 predicted target genes that are currently annotated in Reactome.

Table G in S1 File reports Reactome pathways resulting enriched among predicted targets of each of the considered isomiRs belonging to differentially expressed sRNAs, giving details of pathways most enriched among the targets of specific miRNAs and also indicates that different miRNAs target genes converge to the same enriched pathways, as “Signaling in FGFR in disease”, “DAP12 signaling”, “Oncogene induced senescence” and “Post-transcriptional silencing by small RNAs”.

Human fibroblast grow factor receptors (FGFRs) are a family of four tyrosine kinase receptors (FGFR1–4), which are involved, in a variety of cellular processes. They are indeed key regulators of fibrogenesis, embryogenesis, angiogenesis, metabolism, and many other processes of proliferation and differentiation[72,73]. Deregulation of FGFR signaling has been observed in numerous tumors.[74,75]

DAP12 is an immunoreceptor tyrosine-based activation motif (ITAM)-bearing transmembrane adapter molecule and it is reported to be signaling partner of activating natural killer receptors. DAP12 complex to TREM-1 and MDL-1 receptors to form receptor complexes involved in macrophage differentiation[76] and apoptosis in M1 leukemia cells[77], significant monocytic activation of myeloid cell, calcium mobilization and inflammatory response[78,79]. Its elevated expression levels are associates with enhanced cytotoxic characteristics in large granular lymphocyte leukemia[80].

Senescence is the stable cell growth arrest. Oncogene senescence (OIS) occurs when the activation of an oncogene is triggered; in this case it is termed oncogene-induced senescence. OIS acts as a barrier against tumour progression by driving stable growth arrest of cancer progenitor cells [81–83].

As anticipated, we then considered 3'-moR-128-2 and miR-128-3p targets for comparison. We predicted the targets of the four most expressed miR-128-3p isomiRs (miR-128-3p-exact, miR-128-3p-SL-2, miR-128-3p-SL-3, and miR-128-3p-SL-4) and targets of the two 3'-moR-128-2 isomoRs (3'-moR-128-2-1 and 3'-moR-128-2-2)(Fig 6A), assuming that they would act as miRNA, as indicated by the available experimental data[69,70]. We compared the union of predicted targets of miR-128-3p variants and the union of predicted targets of 3-moR-128-2 variants, to understand if and how much the moRNA function can be related to that of the cognate miRNA, as previously supposed[70]. As shown in the Venn diagram in Fig 6B, only a small fraction of 3-moR-128-2 target genes, less than 17%, is putatively targeted also by at least one of the miR-128-3p isomiRs.

According to Reactome-based functional enrichments, performed as explained in the previous paragraph, different pathways are enriched in predicted targets of 3’-moR-128-2 and of miR-128-3p. miR-128-3p targets are enriched in genes that are part of cellular pathways for the most part related to NGF, FGFR, ERBB4, ERBB2 signaling and transduction and to calcium ion homeostasis and signal transduction.

Targets of 3’-moR-128-2 are enriched too in genes part of several, distinct, pathways related to cellular signaling in growth and proliferation as “Signaling by Notch“, “Signaling by ERBB4”, “Signaling by FGFR in disease” but also, quite interestingly, in genes part of the “Post-transcriptional silencing by small RNAs” and of the more general “Regulatory RNA pathways” (Table H in S1 File).

Remembering that 3’-moR-128-2 is highly expressed in normal and not detected in PMF CD34+, it is worth notice that, 4 out of 7 genes of the “Post-transcriptional silencing by small RNAs” path, namely AGO1, AGO3, TNRC6A, and TNRC6B can be targeted by at least one isomoR of 3-moR-128-2 (Table 3). Moreover, both considered 3-moR-128-2 isomoRs could also target RAN (Table 3), the RAS-related nuclear protein, member of the RAS Oncogene Family, which is required for RNA export from the nucleus (Fig 6C).

**Table 3:** Putative targets of 3’-moR-128-2 variants in the “Regulatory RNA” pathway.

Short RNA variant	Sequence	Target Genes
3'-moR-128-2-1	CCCTACTGTGTCACACTCCTAAT	AGO3—Argonaute 3
3'-moR-128-2-1	CCCTACTGTGTCACACTCCTAAT	RAN—RAN, member RAS oncogene family
3'-moR-128-2-1	CCCTACTGTGTCACACTCCTAAT	POLR2H - Polymerase (RNA) II (DNA directed) polypeptide H
3'-moR-128-2-2	CCTACTGTGTCACACTCCTAAT	AGO1—Argonaute 1
3'-moR-128-2-2	CCTACTGTGTCACACTCCTAAT	RAN—RAN, member RAS oncogene family
3'-moR-128-2-2	CCTACTGTGTCACACTCCTAAT	TNRC6A - Trinucleotide repeat containing 6A
3'-moR-128-2-2	CCTACTGTGTCACACTCCTAAT	TNRC6B - Trinucleotide repeat containing 6B

In principle 3’-moR-128-2, where it is expressed, as in CD34+ hematopoietic stem cells, could affect the expression of genes important for the entire process of miRNA-based silencing. It can indeed target genes essential for post-transcriptional silencing both by translation repression, as AGO1/3, and by mRNA degradation, as TNRC6A/B. AGO1 and AGO3 are required for post-transcriptional translation repression activity; AGO1 is also involved in transcriptional silencing of promoters[84], and AGO3 is also putatively involved into the modulation of mature miRNA incorporation to the RISC complex, thus controlling the ratio between microRNA guide and passenger strand [85]. TNRC6A, and TNRC6B play a role in miRNA-dependent translation repression and endonucleolytic cleavage, by recruiting specific deadenylase complexes.

The multifunctional protein RAN is involved in many processes and diseases: it controls cell cycle progression and it is a potential therapeutic target for treatment of cancers with activation of the PI3K/Akt/mTORC1 and Ras/MEK/ERK pathways[86]. Specifically in relation to the above mentioned findings, as known, RAN play a key role in RNA export from the nucleus and for the biogenesis of all miRNAs. Thus, RAN silencing by 3’-moR-128-2 can impair pre-miRNA transportation to the cytoplasm and output a reduction of miRNA biogenesis. A similar situation was documented by a recent study that identified, in *B*. *mori*, a virus-encoded miRNA that suppresses the host miRNA biogenesis, exactly by targeting the host exportin-5 RAN cofactor [87].

### Validation of potential mRNA targets

Bioinformatics predictions yielded several hundreds putative target genes for each selected small RNA (moR-128-2, miR-379-5p, miR10b-5p, miR-19b-3p, miR-29a-3p and miR-543). Because only a few predicted targets have been experimentally validated in vitro or in vivo, and in order to narrow the analysis to a manageable number of variables, we choose to focus on possible targets eventually selected based on their potential pathogenetic role in PMF.

TaqMan assays were carried out for 18 putative target genes in an independent cohort of CD34+ cells from 20 PMF patients and 10 healthy subjects (Table 4). Using real-time RT-PCR, we found that TNSF10, MME, TCF4, TRPS1 and SYS1 were all significantly reduced in PMF CD34+ compared with healthy controls, whereas BRCA1 and FKBP10 were increased. Other genes, probably due to the limited number of samples, did not reach a statistically significant difference yet they showed the expected trend of expression changes. In particular, we found that MECOM, MEIS1, AGO1, AGO3, RAN, CAV1, AKR1C1, TIMP3, FSTL1 were increased in CD34+ of PMF patients compared with normal subjects, while JAKMIP2 and PTPN4 were reduced.

**Table 4:** Expression pattern of 18 mRNAs potentially targeted by miRNAs, according to qRT-PCR data. For each mRNA, miRNA and moRNA, we report if it is increased (I) or decreased (D) in the PMF VS CTR sample comparison; the mRNAs p-values associated to a significant difference are listed; the last column indicates if the expression of observed target mRNA variation is inversely related to the corresponding miRNA or moRNA.

miRNA name	miRNA I/D	mRNA target gene name	mRNA I/D	mRNA DE p-value	Opposite variation
moR-128-2	D	MECOM	I	NS	YES
moR-128-2	D	MEIS1	I	NS	YES
moR-128-2	D	AGO1	I	NS	YES
moR-128-2	D	AGO3	I	NS	YES
moR-128-2	D	RAN	I	NS	YES
moR-128-2	D	CAV1	I	NS	YES
moR-128-2	D	TNSF10	D	0.0001	NO
miR-379-5p	I	MME	D	0.0001	YES
miR-379-5p	I	SYS1	D	0.0005	YES
miR-379-5p	I	TCF4	D	0.05	YES
miR-379-5p	I	BRCA1	I	0.05	NO
miR-379-5p	I	AKR1C1	I	NS	NO
miR-379-5p	I	TIMP3	I	NS	NO
miR-19b-3p	I	TRPS1	D	0.04	YES
miR-19b-3p	I	PTPN4	D	NS	YES
miR-19b-3p	I	FKBP10	I	0.02	NO
miR-29a-3p	I	AGO3	I	NS	NO
miR-543	I	TRPS1	D	0.04	YES
miR-10b-5p	D	FSTL1	I	NS	YES
miR-10b-5p	D	AGO3	I	NS	YES
miR-10b-5p	D	JAKMIP2	D	NS	NO
miR-10b-5p	D	PTPN4	D	NS	NO

According to miRNAs expression levels, and considering only significantly differentially expressed genes, an opposite behavior of expression variation was found for miR-379-5p and MME, TCF4 and SYS1; miR-19b-3p and miR-543 with TRPS1. Moreover, miR-10b-5p and FSTL1 showed opposite behavior and 5 tested genes (AGO1, RAN, MECOM, MEIS1, and CAV1) putative targets of moR-128-2 showed a trend toward increase in PMF, opposite to the moRNA.

Table 4 reports results of mRNA expression tests, indicating, for each miRNA-mRNA pair, if the observed variations are in the same or in the opposite sense. However, before one can reliably conclude that these predicted interactions do play a role in PMF cell abnormalities and disease pathogenesis, functional studies of miRNA modulation will be required.

## Conclusions

In this study, we reported the results of the first RNA-seq project aiming at studying small RNAs in PMF CD34+ cells compared with control CD34+ cells. We detected 784 know miRNAs expressed in CD34+ cells and discovered 34 new miRNAs produced from known hairpins. We showed that expressed miRNAs with unique sequence are rare and that most miRNAs are isomiR mixtures, whose expression needs to be considered for miRNA expression estimation and for differential expression detection. Moreover, we discovered 99 microRNA-offset RNAs (moRNAs) probably produced from alternative microRNA precursors.

Cluster analyses and heatmaps of samples demonstrated that patients and controls small RNAs profiles are significantly different and highlighted a characteristic miRNA and moRNA expression profile in PMF. In fact, according to RNA-seq data, we identified several sRNAs expressed at significantly different level in patient compared to control CD34+. Subsequent analysis in granulocytes from independent patients and controls samples strengthened the evidence of 6 differentially expressed sRNAs, including 3’-moR-128-2. Predicted targets of these differentially expressed miRNAs are enriched in many remarkable pathways involved in tumor development and progression, as “signaling by FGFR”, “DAP12 signaling” and “Oncogene Induced Senescence”. Moreover, we validated the differential expression of 7 predicted target mRNAs, and identified 5 miRNA-target pairs that show variations in the opposite sense in the PMF vs control CD34+ cell comparison.

We investigated in detail sequence, structure, expression and possible functions of the newly discovered 3'-moR-128-2, also by comparison with the cognate miR-128-3p, showing that the miRNA and moRNA expression profiles are poorly correlated. At the functional level, assuming that moRNAs may act as miRNAs, we noticed that 3'-moR-128-2 and miR-128-3p potential target genes and pathways are markedly different. Interestingly, 3'-moR-128-2, that was expressed in normal CD34+ cells and resulted absent in PMF cells, may target pathways related to the control of cell growth and proliferation and, strikingly, target several genes involved in microRNA biogenesis or in miRNA-mediated silencing.

Overall, in this study, we provided novel data regarding the expression profile of small RNA expressed in PMF CD34+ as well as in healthy control cells, by NGS analysis and their possible impact on specific genes and pathways; in addition, we described for the first time new moRNAs as possible contributors to disease pathogenesis. This information may represent the basis for further studies aimed at a deeper knowledge of the role on miRNAs and moRNAs in normal and pathological hematopoiesis.

## Supporting Information

S1 File Including Tables A-H, Figs A-F and supplementary text with details on target prediction methods. (DOCX)
