# Supplementary material for: Small RNA Sequencing Uncovers New miRNAs and moRNAs Differentially Expressed in Normal and Primary Myelofibrosis CD34+ Cells
Source: PLoS One. 2015 Oct 15;10(10):e0140445. doi: 10.1371/journal.pone.0140445 (PMC4607157; doi:10.1371/journal.pone.0140445)
Supplement: S1 File — (DOCX) [file pone.0140445.s001.docx]

**Supplementary materials**

**Table A.** Metadata for considered CD34+ cells samples (BM, Bone marrow; PB, Peripheral Blood).

| **Sample** | **Raw reads** | **Source** | **Subjects** |
| --- | --- | --- | --- |
| CTR1 | 122,405,675 | BM | 3 Healthy, unknown sex |
| CTR2 | 122,405,675 | BM | 3 Healthy, unknown sex |
| CTR3 | 122,405,675 | BM | 4 Healthy, unknown sex |
| MF1 | 139,926,628 | PB | 4 PMF (#5866, #5939, #5762, #5829), 2 males 2 females |
| MF3 | 128,711,062 | PB | 1 PMF (#5942), male |
| MF4 | 136,210,717 | PB | 1 PMF (#5577) male |

**Figure A.** Effects of different sequence read filtering steps applied to the raw reads to obtain cleaned small RNA-seq samples that were considered for further analyses.

**Figure B.** Samples classification using squared Euclidean distance considering normalized expression profiles of all the small RNAs expressed (A) and considering only those expressed over the median level (B).

| **A**  **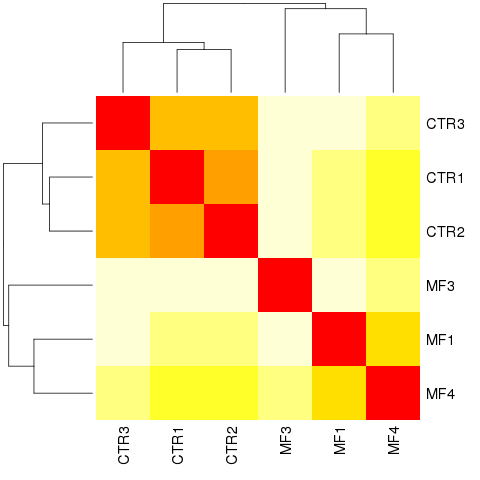** | **B**  **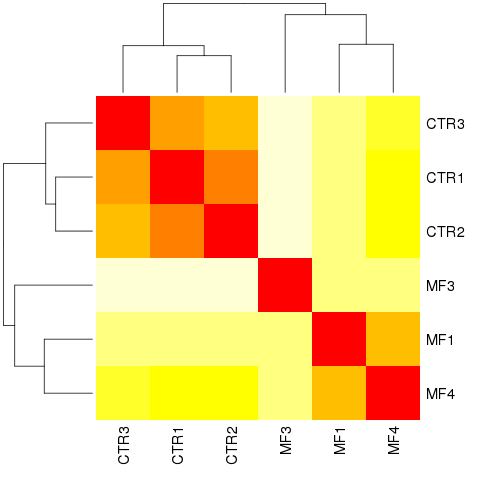** |
| --- | --- |

**Table B.** New miRNAs discovered that are expressed in CD34+. The Table reports expression (per sample group normalized read count), position and sequence of new miRNAs.

| **miRNA** | **Read Count** | | **Strand** | **Position** | **Sequence** |
| --- | --- | --- | --- | --- | --- |
|  | **CTR** | **PMF** |  |  |  |
| hsa-miR-107* | 0 | 19 | - | chr10:89592794-89592815 | AGCTTCTTTACAGTGTTGCCTT |
| hsa-miR-1255a* | 7 | 0 | - | chr4:101330326-101330346 | CTATCTTCTTTGCTCATCCTT |
| hsa-miR-1256* | 13 | 29 | - | chr1:20988343-20988363 | CTAAAGAGAAGTCAATGCATG |
| hsa-miR-1276* | 18 | 0 | - | chr15:85770510-85770530 | TGTCTCCACTGAGCACTTGGG |
| hsa-miR-1284* | 6 | 4 | - | chr3:71542003-71542021 | GAAAGCCCATGTTTGTATT |
| hsa-miR-1289-1* | 12 | 7 | - | chr20:35454037-35454057 | TGCAGACTCTTGGTTTCCACC |
| hsa-miR-1291* | 0 | 10 | - | chr12:48654462-48654490 | ACTGTGGCTGTTGGTTTCAAGCAGAGGCC |
| hsa-miR-1294* | 0 | 2 | + | chr5:154347193-154347213 | ACAGTGCCAACCTCACAGGAC |
| hsa-miR-1302-10* | 0 | 1 | - | chr15:101960531-101960550 | TAGCATAAATATGTCCCAAG |
| hsa-miR-1302-11* | 0 | 1 | + | chr19:72019-72038 | TAGCATAAATATGTCCCAAG |
| hsa-miR-1302-2* | 0 | 1 | + | chr1:30412-30431 | TAGCATAAATATGTCCCAAG |
| hsa-miR-1302-9* | 0 | 1 | + | chr9:30190-30209 | TAGCATAAATATGTCCCAAG |
| hsa-miR-1303* | 0 | 3 | + | chr5:154685789-154685810 | AGCGAGACCTCAACTCTACAAT |
| hsa-miR-153-1-5p | 10 | 19 | - | chr2:219294165-219294185 | GTCATTTTTGTGATCTGCAGC |
| hsa-miR-2110* | 103 | 153 | - | chr10:114174110-114174132 | TCACCGCGGTCTTTTCCTCCCAC |
| hsa-miR-3155a* | 16 | 7 | + | chr10:6152207-6152228 | CCTCCCACTGCAGAGCCTGGGG |
| hsa-miR-3648-1* | 0 | 3 | + | chr21:8208593-8208617 | GTCGGCCGCGCTCGAGGGGTCCCCG |
| hsa-miR-3648-2* | 0 | 3 | + | chr21:8987119-8987143 | GTCGGCCGCGCTCGAGGGGTCCCCG |
| hsa-miR-421* | 0 | 42 | - | chrX:74218427-74218448 | CTCATTAAATGTTTGTTGAATG |
| hsa-miR-4424* | 8 | 21 | + | chr1:178677801-178677821 | GTCCATTTCAAGTTAACTCTG |
| hsa-miR-4473* | 0 | 21 | - | chr9:20411202-20411225 | CACTTGTAATGGAGAACACTAAGC |
| hsa-miR-451a* | 0 | 4 | - | chr17:28861384-28861404 | TTTAGTAATGGTAATGGTTCT |
| hsa-miR-466* | 22 | 0 | - | chr3:31161754-31161774 | TGTGTTGCATGTGTGTATATG |
| hsa-miR-548ag-2* | 28 | 66 | + | chr20:60564601-60564621 | CAAGAACCTCAATTACCTTTG |
| hsa-miR-5696* | 6 | 12 | + | chr2:101309514-101309534 | TCAGACTACCTAAATGAGCAC |
| hsa-miR-599* | 0 | 6 | - | chr8:99536688-99536707 | TTTGATAAGCTGACATGGGA |
| hsa-miR-600* | 5 | 2 | - | chr9:123111598-123111618 | CATAGGAAGGCTCTTGTCTGT |
| hsa-miR-641* | 4 | 6 | - | chr19:40282557-40282576 | TGACTGTCCTATGTCTTTCC |
| hsa-miR-7854-5p | 6 | 3 | + | chr16:81533905-81533926 | CTTCCATCTCCATCACCTTGAG |
| hsa-miR-941-1* | 3 | 2 | + | chr20:63919454-63919478 | ACATGTGCCCAGGGCCCGGGACAGC |
| hsa-miR-941-2* | 3 | 2 | + | chr20:63919510-63919534 | ACATGTGCCCAGGGCCCGGGACAGC |
| hsa-miR-941-3* | 3 | 2 | + | chr20:63919566-63919590 | ACATGTGCCCAGGGCCCGGGACAGC |
| hsa-miR-941-4* | 3 | 2 | + | chr20:63919761-63919785 | ACATGTGCCCAGGGCCCGGGACAGC |
| hsa-miR-941-5* | 3 | 2 | + | chr20:63919873-63919897 | ACATGTGCCCAGGGCCCGGGACAGC |

**Figure C.** Distribution of reads per isomiR category and per type across samples, considering all 818 expressed miRNAs. “Exact” reads are identical to the mature miRNA sequence annotated in miRBase, whereas “mismatch” reads present respectively one or two nucleotides different from the annotated sequence but identical length; the last category includes reads perfecly matching the miRNA precursor (and genomic) sequence but shorter or longer than the annotated mature miRNA. IsomiR types indicate if the sequence difference falls in the 5’ region of the miRNA, in the 3’ region, or in both regions. The figure shows read counts for intersections of considered isomiR categories and types.

**Figure D.** Distribution of reads per isomiR category (A), type (B) across samples, considering 25% most expressed miRNAs (204). “Exact” reads are identical to the mature miRNA sequence annotated in miRBase, whereas “mismatch” reads present respectively one or two nucleotides different from the annotated sequence but identical length; the last category includes reads perfecly matching the miRNA precursor (and genomic) sequence but shorter or longer than the annotated mature miRNA. IsomiR types indicate if the sequence difference fall in the 5’ region of the miRNA, in the 3’ region, or in both regions. Panel C shows read counts for intersections of considered isomiR categories and types.

| **A**  **** | **B**  **** |
| --- | --- |
| **C**  **** | |

**Figure E.** Correlations between expression profiles of isomiRs and of mature miRNA expression, (A) considering, for each miRNA, all isomiRs accounting each for at least 10% of miRNA expression, and (B) focusing on the subset of these isomiRs significantly differentially expressed in CD34+ cells of PMF patients compared to controls (t-test q-value < 0.05).

| **A**   | **B**   |
| --- | --- |

**Table C.** List of moRNAs expressed in considered CD34+ samples. The first seven moRNAs are expressed over the third quartile of all sRNAs expression.

| **moRNA** | **Read count** | | **Strand** | **Position** | **Sequence** |
| --- | --- | --- | --- | --- | --- |
|  | **CTR** | **PMF** |  |  |  |
| hsa-moR-101-1-5p | 0 | 43 | - | chr1:65058500-65058520 | TGACTGACAGGCTGCCCTGGC |
| hsa-moR-103a-1-5p | 0 | 13 | - | chr5:168560965-168560982 | AAGTTTTCTTACTGCCCT |
| hsa-moR-103a-2-3p | 567 | 252 | + | chr20:3917564-3917586 | AAGAACCAAGAATGGGCTGCCCT |
| hsa-moR-103a-2-5p | 117 | 37 | + | chr20:3917484-3917502 | AGCTGCGTCTTTGTGCTTT |
| hsa-moR-106b-3p | 24 | 0 | - | chr7:100093978-100094001 | TCCAGCAGGGCACGCACAGCGTCC |
| hsa-moR-106b-5p | 508 | 112 | - | chr7:100094065-100094084 | CCGCTCCAGCCCTGCCGGGG |
| hsa-moR-10a-5p | 0 | 11 | - | chr17:48579928-48579946 | ATCTGTCTGTCTTCTGTAT |
| hsa-moR-1248-3p | 0 | 12 | + | chr3:186786787-186786804 | AAAGACTGGGGTGGACCT |
| hsa-moR-125a-5p | 11 | 65 | + | chr19:51693247-51693266 | ACCATGTTGCCAGTCTCTAG |
| hsa-moR-126-5p | 27 | 8 | + | chr9:136670596-136670614 | CGCCTCCGCTGGCGACGGG |
| hsa-moR-128-1-5p | 0 | 4 | + | chr2:135665391-135665409 | TGTTCCTGAGCTGTTGGAT |
| hsa-moR-128-2-3p | 2493 | 0 | + | chr3:35744548-35744568 | CCCTACTGTGTCACACTCCTA |
| hsa-moR-136-5p | 0 | 18 | + | chr14:100884694-100884714 | GTGTTGGATGAGCCCTCGGAG |
| hsa-moR-140-5p | 18 | 10 | + | chr16:69933083-69933101 | TGTCTCTCTCTGTGTCCTG |
| hsa-moR-141-5p | 0 | 14 | + | chr12:6964093-6964111 | CTGTCGGCCGGCCCTGGGT |
| hsa-moR-142-3p | 32 | 13 | - | chr17:58331218-58331244 | TGAGTGTACTGTGGGCTTCGGAGATCA |
| hsa-moR-142-5p | 0 | 27 | - | chr17:58331305-58331332 | CCGACGGACAGACAGACAGTGCAGTCAC |
| hsa-moR-146b-5p | 0 | 3 | + | chr10:102436498-102436518 | AAGAACTTTGGCCACCTGGCA |
| hsa-moR-150-3p | 59 | 4 | - | chr19:49500772-49500797 | GGGACCTGGGGACCCCGGCACCGGCA |
| hsa-moR-154-5p | 0 | 2 | + | chr14:101059749-101059767 | TAGCGTGTGGTACTTGAAG |
| hsa-moR-155-5p | 0 | 14 | + | chr21:25573963-25573981 | TTGCTGTAGGCTGTATGCT |
| hsa-moR-15a-5p | 0 | 3 | - | chr13:50049190-50049208 | AATAAAACCTTGGAGTAAA |
| hsa-moR-15b-5p | 46 | 57 | + | chr3:160404586-160404605 | TTTTGAGGCCTTAAAGTACT |
| hsa-moR-16-1-5p | 263 | 424 | - | chr13:50049050-50049069 | ATAGCAATGTCAGCAGTGCC |
| hsa-moR-16-2-5p | 47 | 31 | + | chr3:160404733-160404752 | ACTGACATACTTGTTCCACT |
| hsa-moR-17-5p | 0 | 4 | + | chr13:91350596-91350616 | TTGTGACCAGTCAGAATAATG |
| hsa-moR-181a-2-5p | 0 | 2 | + | chr9:124692461-124692478 | CCTTCAGAGGACTCCAAG |
| hsa-moR-18a-5p | 9 | 32 | + | chr13:91350736-91350754 | ATGTTGAGTGCTTTTTGTT |
| hsa-moR-196b-5p | 0 | 5 | - | chr7:27169551-27169571 | GCACCAGAACTGGTCGGTGAT |
| hsa-moR-19a-5p | 0 | 16 | + | chr13:91350884-91350902 | TTTGTTTGCAGTCCTCTGT |
| hsa-moR-19b-1-5p | 0 | 16 | + | chr13:91351185-91351205 | TACTGAACACTGTTCTATGGT |
| hsa-moR-20a-5p | 0 | 44 | + | chr13:91351049-91351070 | TGATGTGACAGCTTCTGTAGCA |
| hsa-moR-214-5p | 0 | 49 | - | chr1:172138879-172138896 | ACAGAGTTGTCATGTGTC |
| hsa-moR-21-5p | 2946 | 2437 | + | chr17:59841243-59841271 | ACATCTCCATGGCTGTACCACCTTGTCGG |
| hsa-moR-222-5p | 0 | 9 | - | chrX:45747098-45747116 | AAGGTGTAGGTACCCTCAA |
| hsa-moR-23a-3p | 246 | 98 | - | chr19:13836566-13836592 | CCGACCCTGAGCTCTGCCACCGAGGAT |
| hsa-moR-24-1-5p | 0 | 6 | + | chr9:95086004-95086024 | TGTCGATTGGACCCGCCCTCC |
| hsa-moR-24-2-5p | 5971 | 1719 | - | chr19:13836350-13836376 | TGCCTGGCCTCCCTGGGCTCTGCCTCC |
| hsa-moR-25-5p | 37 | 40 | - | chr7:100093631-100093651 | CCGGGACTGGCCAGTGTTGAG |
| hsa-moR-26a-2-5p | 13 | 27 | - | chr12:57824681-57824699 | CCATAGAGGCTGTGGCTGG |
| hsa-moR-27a-5p | 1752 | 312 | - | chr19:13836510-13836534 | CGAAGCCTGTGCCTGGCCTGAGGAG |
| hsa-moR-29c-5p | 0 | 2 | - | chr1:207801924-207801942 | CCCATCTCTTACACAGGCT |
| hsa-moR-301a-5p | 0 | 3 | - | chr17:59151210-59151230 | CTACTTATTACTGCTAACGAA |
| hsa-moR-30c-1-5p | 0 | 7 | + | chr1:40757279-40757298 | CTATAACCATGCTGTAGTGT |
| hsa-moR-324-5p | 67 | 47 | - | chr17:7223366-7223384 | CTGAGCTGACTATGCCTCC |
| hsa-moR-32-5p | 24 | 19 | - | chr9:109046295-109046313 | TCTGCTTGCTCTGGTGGAG |
| hsa-moR-326-5p | 165 | 561 | - | chr11:75335169-75335193 | CCGGAGCCTCATCTGTCTGTTGGGC |
| hsa-moR-331-5p | 20 | 38 | + | chr12:95308425-95308443 | TGGTTTTGTTTGGGTTTGT |
| hsa-moR-338-5p | 0 | 4 | - | chr17:81125946-81125963 | CCGCACGGCTGTCCTCTC |
| hsa-moR-361-5p | 4 | 21 | - | chrX:85903704-85903722 | TTTTCCTGGGATTTGGGAG |
| hsa-moR-3651-5p | 0 | 1266 | - | chr9:92292537-92292565 | ATGGACAGCTCTCCAGTGGATTCGATGGG |
| hsa-moR-370-5p | 0 | 18 | + | chr14:100911130-100911149 | CGGGGCACAAGACAGAGAAG |
| hsa-moR-374a-5p | 0 | 39 | - | chrX:74287348-74287366 | AAGAAATTTTACATCGGCC |
| hsa-moR-377-5p | 0 | 49 | + | chr14:101062031-101062055 | CCGTGCTGATGTTTGACCCTTGAGC |
| hsa-moR-378a-5p | 6 | 4 | + | chr5:149732810-149732827 | GTGACAGAGCCACCCAGG |
| hsa-moR-382-5p | 0 | 33 | + | chr14:101054296-101054314 | TTTTCTGTGGTACTTGAAG |
| hsa-moR-421-3p | 0 | 2 | - | chrX:74218372-74218391 | CTGCTCTGTGATCTCCATGG |
| hsa-moR-421-5p | 848 | 2784 | - | chrX:74218449-74218472 | CCTAATCCGGTGCACATTGTAGGC |
| hsa-moR-4485-5p | 0 | 17 | - | chr11:10508315-10508332 | AGTATTAGAGGCACCGCC |
| hsa-moR-4521-5p | 24 | 5 | + | chr17:8186930-8186948 | CGAATCCCATCCTCGTCGG |
| hsa-moR-493-5p | 0 | 3 | + | chr14:100869048-100869073 | TCGGGGCTCATTCTGGCCTCCAGGGC |
| hsa-moR-496-5p | 0 | 5 | + | chr14:101060575-101060594 | CAAGTCAGGTACTCGAATGG |
| hsa-moR-503-5p | 18 | 32 | - | chrX:134546395-134546413 | GCCCGCGCTCAGCCGTGCC |
| hsa-moR-505-5p | 0 | 6 | - | chrX:139924219-139924237 | TAAATTGATGCACCCAGTG |
| hsa-moR-542-5p | 9 | 237 | - | chrX:134541424-134541442 | ATGCACAGATCTCAGACAT |
| hsa-moR-545-5p | 0 | 24 | - | chrX:74287189-74287208 | CCAGCCTGGCACATTAGTAG |
| hsa-moR-548b-5p | 0 | 2 | - | chr6:119069121-119069138 | TATATATTTAGGTTGGCG |
| hsa-moR-551b-3p | 11 | 41 | + | chr3:168551935-168551962 | AGGCTGTGAGAATAACTGCAATTTAGAG |
| hsa-moR-551b-5p | 4 | 15 | + | chr3:168551855-168551873 | GATGTGCTCTCCTGGCCCA |
| hsa-moR-5695-3p | 0 | 7 | + | chr19:12920384-12920403 | AATCTAGACAGATAGGCCTT |
| hsa-moR-6087-3p | 0 | 2 | + | chrX:109054577-109054601 | CTTCTGGCGCCAAGCGCCCGGCCGC |
| hsa-moR-625-5p | 0 | 2 | + | chr14:65471097-65471114 | TGGTAAGGGTAGAGGGAT |
| hsa-moR-671-5p | 5 | 0 | + | chr7:151238430-151238447 | CTGGCAGGCCAGGAAGAG |
| hsa-moR-6724-1-5p | 683 | 280 | + | chr21:8205298-8205332 | TGTGGGGGAGAGGCTGTCGCTGCGCTTCTGGGCCC |
| hsa-moR-6724-2-5p | 683 | 280 | + | chr21:8249488-8249522 | TGTGGGGGAGAGGCTGTCGCTGCGCTTCTGGGCCC |
| hsa-moR-6724-3-5p | 683 | 280 | + | chr21:8388345-8388379 | TGTGGGGGAGAGGCTGTCGCTGCGCTTCTGGGCCC |
| hsa-moR-6724-4-5p | 683 | 280 | + | chr21:8432513-8432547 | TGTGGGGGAGAGGCTGTCGCTGCGCTTCTGGGCCC |
| hsa-moR-7-1-5p | 213 | 171 | - | chr9:83969836-83969859 | CATTGGATGTTGGCCTAGTTCTGT |
| hsa-moR-766-5p | 0 | 4 | - | chrX:119646822-119646841 | CAGGACCTGGGCTTGGGTGG |
| hsa-moR-769-5p | 171 | 218 | + | chr19:46018939-46018959 | TGCTGATTCCTGGGCTCTGAC |
| hsa-moR-876-5p | 0 | 9 | - | chr9:28863698-28863716 | ACACAAACTGTGAAGTGCT |
| hsa-moR-92a-1-5p | 6 | 32 | + | chr13:91351303-91351322 | AACTCAAACCCCTTTCTACA |
| hsa-moR-93-5p | 0 | 33 | - | chr7:100093839-100093861 | CTTGGACCTCAGTCCTGGGGGCT |
| hsa-moR-941-1-3p | 3 | 2 | + | chr20:63919510-63919534 | ACATGTGCCCAGGGCCCGGGACAGC |
| hsa-moR-941-2-3p | 3 | 2 | + | chr20:63919566-63919590 | ACATGTGCCCAGGGCCCGGGACAGC |
| hsa-moR-941-3-3p | 3 | 2 | + | chr20:63919622-63919646 | ACATGTGCCCAGGGCCCGGGACAGC |
| hsa-moR-941-4-3p | 3 | 2 | + | chr20:63919817-63919841 | ACATGTGCCCAGGGCCCGGGACAGC |
| hsa-moR-941-4-5p | 6564 | 2531 | + | chr20:63919746-63919768 | CACCCGGCTGTGTGCACATGTGC |
| hsa-moR-941-5-3p | 3 | 2 | + | chr20:63919929-63919953 | ACATGTGCCCAGGGCCCGGGACAGC |
| hsa-moR-941-5-5p | 9780 | 3800 | + | chr20:63919858-63919880 | CACCCGGCTGTGTGCACATGTGC |
| hsa-moR-98-5p | 0 | 9 | - | chrX:53556322-53556340 | GGATTCTGCTCATGCCAGG |
| hsa-moR-99b-5p | 0 | 2 | + | chr19:51692594-51692616 | CCCGGACTCCTGGGTCCTGGCAC |
| hsa-moR-let-7a-1-5p | 27 | 4 | + | chr9:94175942-94175960 | ATGTTCTCTTCACTGTGGG |
| hsa-moR-let-7a-2-5p | 0 | 4 | - | chr11:122146591-122146611 | TTGTGACTGCATGCTCCCAGG |
| hsa-moR-let-7a-3-5p | 0 | 2 | + | chr22:46112732-46112750 | AGACCGACTGCCCTTTGGG |
| hsa-moR-let-7b-5p | 9 | 27 | + | chr22:46113671-46113689 | CAAGGCCGGGCCTGGCGGG |
| hsa-moR-let-7d-5p | 15 | 18 | + | chr9:94178821-94178839 | AAAAAAATGGGTTCCTAGG |
| hsa-moR-let-7f-2-5p | 22 | 46 | - | chrX:53557269-53557287 | GACACTGGTGCTCTGTGGG |
| hsa-moR-let-7i-5p | 31 | 37 | + | chr12:62603669-62603689 | TCCCCGACACCATGGCCCTGG |

**Table D.** miRNAs and moRNAs significantly differentially expressed (DE) in PMF patient respect to control CD34+, according to RNA-seq data.

| **sRNA** | **Average normalized read count** | | **log2(FC)** | **p-value** | **Adjusted p-value** |
| --- | --- | --- | --- | --- | --- |
|  | **CTR** | **PMF** |  |  |  |
| hsa-miR-1185-5p | 0 | 136 | 15.00 | 1.24E-05 | 6.98E-04 |
| hsa-miR-127-5p | 0 | 317 | 15.00 | 4.66E-07 | 7.01E-05 |
| hsa-miR-1277-5p | 0 | 98 | 15.00 | 1.53E-04 | 6.28E-03 |
| hsa-miR-299-3p | 0 | 210 | 15.00 | 1.78E-04 | 6.69E-03 |
| hsa-miR-323a-3p | 0 | 121 | 15.00 | 5.34E-03 | 7.29E-02 |
| hsa-miR-377-3p | 0 | 206 | 15.00 | 9.34E-04 | 1.92E-02 |
| hsa-miR-377-5p | 0 | 155 | 15.00 | 8.32E-06 | 6.25E-04 |
| hsa-miR-379-3p | 0 | 89 | 15.00 | 2.76E-04 | 9.56E-03 |
| hsa-miR-382-5p | 0 | 205 | 15.00 | 2.97E-04 | 9.56E-03 |
| hsa-miR-431-3p | 0 | 80 | 15.00 | 1.72E-03 | 3.11E-02 |
| hsa-miR-490-3p | 0 | 719 | 15.00 | 2.48E-03 | 3.99E-02 |
| hsa-miR-539-3p | 0 | 204 | 15.00 | 1.37E-03 | 2.58E-02 |
| hsa-miR-543 | 0 | 262 | 15.00 | 4.64E-04 | 1.40E-02 |
| hsa-miR-654-5p | 0 | 99 | 15.00 | 6.37E-04 | 1.60E-02 |
| hsa-miR-656 | 0 | 78 | 15.00 | 8.90E-04 | 1.91E-02 |
| hsa-miR-665 | 0 | 317 | 15.00 | 2.67E-03 | 4.15E-02 |
| hsa-miR-758-3p | 0 | 171 | 15.00 | 3.04E-03 | 4.56E-02 |
| hsa-miR-873-5p | 0 | 211 | 15.00 | 6.57E-04 | 1.60E-02 |
| hsa-miR-25-3p | 37 | 226192 | 12.59 | 2.85E-27 | 1.29E-24 |
| hsa-miR-29a-3p | 206 | 44817 | 7.77 | 6.73E-04 | 1.60E-02 |
| hsa-miR-136-5p | 34 | 1516 | 5.47 | 1.24E-04 | 5.57E-03 |
| hsa-miR-495-3p | 8 | 281 | 5.09 | 6.43E-04 | 1.60E-02 |
| hsa-miR-873-3p | 7 | 239 | 5.04 | 5.15E-03 | 7.27E-02 |
| hsa-miR-485-5p | 12 | 353 | 4.84 | 1.17E-03 | 2.30E-02 |
| hsa-miR-19b-3p | 3795 | 99173 | 4.71 | 7.13E-07 | 8.03E-05 |
| hsa-miR-432-5p | 14 | 343 | 4.62 | 2.09E-03 | 3.62E-02 |
| hsa-5'-moR-542 | 9 | 232 | 4.62 | 6.72E-03 | 8.66E-02 |
| hsa-miR-379-5p | 31 | 561 | 4.16 | 7.86E-03 | 9.58E-02 |
| hsa-miR-19a-5p | 15 | 265 | 4.16 | 5.16E-03 | 7.27E-02 |
| hsa-miR-33b-5p | 26 | 394 | 3.90 | 6.09E-03 | 8.08E-02 |
| hsa-miR-1307-5p | 779 | 10914 | 3.81 | 8.23E-04 | 1.86E-02 |
| hsa-miR-142-3p | 5954 | 33867 | 2.51 | 7.73E-03 | 9.58E-02 |
| hsa-miR-3150b-3p | 196 | 7 | -4.90 | 2.23E-03 | 3.73E-02 |
| hsa-miR-10b-5p | 119504 | 2855 | -5.39 | 2.15E-08 | 4.84E-06 |
| hsa-3'-moR-128-2 | 2489 | 0 | -15.00 | 1.15E-05 | 6.98E-04 |
| hsa-miR-128-2* | 102 | 0 | -15.00 | 1.87E-05 | 9.36E-04 |
| hsa-miR-5008-3p | 149 | 0 | -15.00 | 4.17E-06 | 3.76E-04 |

**Figure F.** Expression variations, according to RT-PCR quantifications, of five miRNAs and moR-128-2 in granulocytes collected from an independent cohort of normal controls (N=10) and of PMF (N=50), PV (N=30) or ET (N=30) patients.

Target prediction of small RNAs deregulated in PMF

miRNAs bind to mRNAs by Watson-Crick pairing of miRNA nucleotides 2-8, called “seed region”, to a short region of mRNA termed miRNA recognition elements (MREs), generally situated at 3’ untraslated region (UTR). Rules that govern miRNA recognizing its target are not fully understood and the mechanism is poorly characterized. The binding is not necessary a perfect base pairing and the MRE is not located in a fixed position, easily identifiable along the mRNA sequence. What’s more, there can be multiple binding sites along the same mRNA. Since seed region is very limited, there are numerous predicted MREs sequences that match; so considering only sequence complementarity in the searching for MREs produces thousands of false positives. The complexity of miRNA-mRNA interactions causes ambiguity in target prediction results. Target genes identification is indeed challenging and many algorithms have been developed. Target prediction programs can be divided in two classes, distinguished on the basis of the use or not of the information about evolutionary conservation of interaction. We choose to perform a target prediction using two different programs, miRanda and PITA , which implement orthogonal target prediction strategies. Our choice was determined also by code availability, which allowed us to make custom predictions using as query sequences also isomiRs and moRNA sequences.

Among different isomiRs detected for each considered miRNA, we considered the most expressed. We also included those variants that were significantly contributing to miRNA total expression and which were differently expressed in patients respect to controls (t-test<0.05 and |log2FC| > 1).

We performed a hypergeometric test using an in-house modified version of the R Category package of Bioconductor, which supports Reactome annotation maps via the reactome.db R package.

Table E. The table shows miRNA and moRNA sequences considered for target prediction and corresponding results, in terms of number of targets predicted by each algorithm, and predicted by both considered methods.

| **IsomiR** | **Sequence** | **Category** | **miRanda** | **Pita** | **Common** |
| --- | --- | --- | --- | --- | --- |
| hsa-3p-moR-128-2 | CCTACTGTGTCACACTCCTAAT | exact | 3254 | 992 | 578 |
| hsa-miR-10b-5p-FC_T | ACCCTGTAGAACCGAATTTGT | shorter-longer | 3503 | 909 | 542 |
| hsa-miR-10b-5p-HIGHER | TACCCTGTAGAACCGAATTTGT | shorter-longer | 3112 | 433 | 252 |
| hsa-miR-19b-3p-HIGHER | TGTGCAAATCCATGCAAAACTG | shorter-longer | 4340 | 454 | 364 |
| hsa-miR-29a-3p-EXACT_HIGHER | TAGCACCATCTGAAATCGGTTA | exact | 4052 | 659 | 473 |
| hsa-miR-29a-3p-FC_T | CTAGCACCATCTGAAATCGGTT | shorter-longer | 2645 | 692 | 418 |
| hsa-miR-379-5p-EXACT_HIGHER | TGGTAGACTATGGAACGTAGG | exact | 1993 | 2222 | 861 |
| hsa-miR-543-EXACT_HIGHER | AAACATTCGCGGTGCACTTCTT | exact | 3993 | 495 | 347 |

Table F. Reactome pathways significantly (q-value <= 0.05) enriched among the union of predicted target genes of miRNAs and moRNAs differentially expressed in PMF vs CTR samples.

| **Reactome Pathway** | | **Gene symbols** | **Obs. count** | **Exp. count** | **Fold enrich.** | **p-value** | **q-value** |
| --- | --- | --- | --- | --- | --- | --- | --- |
| **ID** | **Name** |  |  |  |  |  |  |
| 1963642 | PI3K events in ERBB2 signaling | AKT2, AKT3, FOXO1, FOXO4, GSK3B, MDM2, TSC2, GAB1, EGFR, ERBB4, EREG, NRG1, NRG4, NRG2, ERBB3, TNRC6A, TNRC6B, AGO2, AGO1, AGO3, THEM4, PTEN, PHLPP2, PIK3CA, TRAT1, KIT, IRS1, IRS2, FRS2, CD86, CD28, PIK3CB, FGFR1, KLB, FGF1, FGF2, FGF10, RICTOR | 38 | 22.3 | 1.71 | 0.0002 | 0.0147 |
| 1250342 | PI3K events in ERBB4 signaling | AKT2, AKT3, FOXO1, FOXO4, GSK3B, MDM2, TSC2, ERBB4, EREG, NRG1, NRG4, NRG2, PIK3CA, TNRC6A, TNRC6B, AGO2, AGO1, AGO3, THEM4, PTEN, PHLPP2, TRAT1, KIT, IRS1, IRS2, GAB1, FRS2, CD86, CD28, PIK3CB, EGFR, FGFR1, KLB, ERBB3, FGF1, FGF2, FGF10, RICTOR | 38 | 22.3 | 1.71 | 0.0002 | 0.0147 |
| 2219528 | PI3K/AKT Signaling in Cancer | AKT2, AKT3, FOXO1, FOXO4, GSK3B, MDM2, TSC2, RICTOR, PIK3CA, PTEN, TRAT1, KIT, IRS1, IRS2, GAB1, FRS2, CD86, CD28, PIK3CB, EGFR, FGFR1, KLB, ERBB3, ERBB4, FGF1, FGF2, FGF10, NRG2, NRG1, EREG, NRG4, TNRC6A, TNRC6B, AGO2, AGO1, AGO3, THEM4, PHLPP2 | 38 | 22.3 | 1.71 | 0.0002 | 0.0147 |
| 1257604 | PIP3 activates AKT signaling | AKT2, AKT3, FOXO1, FOXO4, GSK3B, MDM2, TSC2, TNRC6A, TNRC6B, AGO2, AGO1, AGO3, THEM4, PTEN, PHLPP2, PIK3CA, TRAT1, KIT, IRS1, IRS2, GAB1, FRS2, CD86, CD28, PIK3CB, EGFR, FGFR1, KLB, ERBB3, ERBB4, FGF1, FGF2, FGF10, NRG2, NRG1, EREG, NRG4, RICTOR | 38 | 22.3 | 1.71 | 0.0002 | 0.0147 |
| 190236 | Signaling by FGFR | FRS2, FGFR1, KLB, MAPK1, FGF1, FGF2, FGF10, SHC1, RAF1, ADCY2, ADCY6, ADCY1, AKT2, AKT3, FOXO1, FOXO4, GSK3B, MDM2, TSC2, BRAF, GAB1, PDE1C, CAMK4, PRKACB, PRKCA, PRKAR2A, CBL, ITPR3, ITPR1, ITPR2, PIK3CA, SOS1, PTEN, RICTOR, THEM4, TRAT1, TNRC6A, TNRC6B, AGO2, KIT, IRS1, IRS2, PHLPP2, AGO1, AGO3, CD86, CD28, PIK3CB, EGFR, ERBB3, ERBB4, NRG2, NRG1, EREG, NRG4, MKNK1 | 56 | 34.9 | 1.60 | 6.49998E-05 | 0.0147 |
| 187037 | NGF signalling via TRKA from the plasma membrane | BRAF, KIDINS220, FRS2, RAF1, ADCYAP1, NTRK2, IRS1, IRS2, PIK3CA, PIK3CB, ADCY2, ADCY6, ADCY1, AKT2, AKT3, FOXO1, FOXO4, GSK3B, MDM2, TSC2, MAPK1, DNAL4, AP2A1, PDE1C, CAMK4, PRKACB, PRKCA, PRKAR2A, RPS6KA5, RPS6KA2, ITPR3, ITPR1, ITPR2, DNM3, ELK1, MEF2C, PPP2R5D, PPP2R1B, DUSP6, SOS1, SHC1, TNRC6A, TNRC6B, AGO2, AGO1, AGO3, THEM4, PTEN, PHLPP2, TRAT1, KIT, GAB1, CD86, CD28, EGFR, FGFR1, KLB, ERBB3, ERBB4, FGF1, FGF2, FGF10, NRG2, NRG1, EREG, NRG4, RICTOR, RIT1 | 68 | 45.7 | 1.49 | 0.0002 | 0.0147 |
| 1643713 | Signaling by EGFR in Cancer | RAF1, GAB1, EGFR, ADCY2, ADCY6, ADCY1, AKT2, AKT3, FOXO1, FOXO4, GSK3B, MDM2, TSC2, EPS15L1, CBL, AP2A1, STAM2, CDC42, SOS1, PIK3CA, SHC1, PDE1C, CAMK4, PRKACB, PRKCA, PRKAR2A, ITPR3, ITPR1, ITPR2, MAPK1, PTEN, RICTOR, THEM4, TRAT1, TNRC6A, TNRC6B, AGO2, KIT, IRS1, IRS2, PHLPP2, AGO1, AGO3, FRS2, CD86, CD28, PIK3CB, FGFR1, KLB, ERBB3, ERBB4, FGF1, FGF2, FGF10, NRG2, NRG1, EREG, NRG4, ADAM12 | 59 | 39.0 | 1.51 | 0.0003 | 0.0173 |
| 198203 | PI3K/AKT activation | IRS1, IRS2, PIK3CA, PIK3CB, AKT2, AKT3, FOXO1, FOXO4, GSK3B, MDM2, TSC2, TNRC6A, TNRC6B, AGO2, AGO1, AGO3, THEM4, PTEN, PHLPP2, TRAT1, KIT, GAB1, FRS2, CD86, CD28, EGFR, FGFR1, KLB, ERBB3, ERBB4, FGF1, FGF2, FGF10, NRG2, NRG1, EREG, NRG4, RICTOR | 38 | 23.0 | 1.66 | 0.0005 | 0.0262 |
| 2172127 | DAP12 interactions | RAF1, VAV2, GRAP2, PLCG2, SHC1, SOS1, ADCY2, ADCY6, ADCY1, AKT2, AKT3, FOXO1, FOXO4, GSK3B, MDM2, TSC2, PDE1C, CAMK4, PRKACB, PRKCA, PRKAR2A, ITPR3, ITPR1, ITPR2, PIK3CA, PIK3CB, PTEN, KLRK1, RICTOR, THEM4, MAPK1, TRAT1, TNRC6A, TNRC6B, AGO2, KIT, IRS1, IRS2, GAB1, PHLPP2, AGO1, AGO3, FRS2, CD86, CD28, EGFR, FGFR1, KLB, ERBB3, ERBB4, FGF1, FGF2, FGF10, NRG2, NRG1, EREG, NRG4, KIR2DL2, KIR2DS5, KIR2DS1, KIR3DL3, KIR2DL3, KIR2DL1, KIR2DS4, CLEC5A | 65 | 39.0 | 1.67 | 4.14939E-06 | 0.0054 |
| 186797 | Signaling by PDGF | RAF1, ADCY2, ADCY6, ADCY1, AKT2, AKT3, FOXO1, FOXO4, GSK3B, MDM2, TSC2, PDE1C, CAMK4, PRKACB, PRKCA, PRKAR2A, ITPR3, ITPR1, ITPR2, MAPK1, BCAR1, SOS1, STAT1, STAT6, PIK3CA, PIK3CB, PTEN, RICTOR, THEM4, TRAT1, TNRC6A, TNRC6B, AGO2, KIT, IRS1, IRS2, GAB1, PHLPP2, AGO1, AGO3, FRS2, CD86, CD28, EGFR, FGFR1, KLB, ERBB3, ERBB4, FGF1, FGF2, FGF10, NRG2, NRG1, EREG, NRG4, PDGFC, PDGFD, THBS2, COL4A1, COL4A3, COL4A4, COL4A5, COL6A3, COL2A1, COL3A1, COL5A1, COL5A2 | 67 | 41.3 | 1.62 | 8.60918E-06 | 0.0056 |
| 1226099 | Signaling by FGFR in disease | FRS2, FGFR1, KLB, MAPK1, FGF1, FGF2, FGF10, SHC1, RAF1, ADCY2, ADCY6, ADCY1, AKT2, AKT3, FOXO1, FOXO4, GSK3B, MDM2, TSC2, BRAF, PIK3CA, GAB2, GAB1, PDE1C, CAMK4, PRKACB, PRKCA, PRKAR2A, CBL, ITPR3, ITPR1, ITPR2, FGFR1OP, LRRFIP1, MYO18A, TRIM24, SOS1, PTEN, RICTOR, THEM4, TRAT1, TNRC6A, TNRC6B, AGO2, KIT, IRS1, IRS2, PHLPP2, AGO1, AGO3, CD86, CD28, PIK3CB, EGFR, ERBB3, ERBB4, NRG2, NRG1, EREG, NRG4, STAT1, MKNK1 | 62 | 38.3 | 1.62 | 2.03827E-05 | 0.0088 |
| 190926 | PI-3K cascade | AKT2, AKT3, FOXO1, FOXO4, GSK3B, MDM2, TSC2, GAB1, PIK3CA, FRS2, FGFR1, KLB, FGF1, FGF2, FGF10, TNRC6A, TNRC6B, AGO2, AGO1, AGO3, THEM4, PTEN, PHLPP2, TRAT1, KIT, IRS1, IRS2, CD86, CD28, PIK3CB, EGFR, ERBB3, ERBB4, NRG2, NRG1, EREG, NRG4, RICTOR | 38 | 22.3 | 1.71 | 0.0002 | 0.0147 |
| 2730905 | Role of LAT2/NTAL/LAB on calcium mobilization | AKT2, AKT3, FOXO1, FOXO4, GSK3B, MDM2, TSC2, TNRC6A, TNRC6B, AGO2, AGO1, AGO3, THEM4, PTEN, PHLPP2, GAB2, SHC1, SOS1, PIK3CA, PIK3CB, TRAT1, KIT, IRS1, IRS2, GAB1, FRS2, CD86, CD28, EGFR, FGFR1, KLB, ERBB3, ERBB4, FGF1, FGF2, FGF10, NRG2, NRG1, EREG, NRG4, RICTOR | 41 | 24.3 | 1.69 | 0.0002 | 0.0147 |
| 190333 | Downstream signaling of activated FGFR | FRS2, FGFR1, KLB, FGF1, FGF2, FGF10, SHC1, RAF1, ADCY2, ADCY6, ADCY1, AKT2, AKT3, FOXO1, FOXO4, GSK3B, MDM2, TSC2, GAB1, PDE1C, CAMK4, PRKACB, PRKCA, PRKAR2A, ITPR3, ITPR1, ITPR2, MAPK1, PIK3CA, SOS1, TNRC6A, TNRC6B, AGO2, AGO1, AGO3, THEM4, PTEN, PHLPP2, RICTOR, TRAT1, KIT, IRS1, IRS2, CD86, CD28, PIK3CB, EGFR, ERBB3, ERBB4, NRG2, NRG1, EREG, NRG4 | 53 | 32.6 | 1.63 | 6.79885E-05 | 0.0147 |
| 112314 | Neurotransmitter Receptor Binding And Downstream Transmission In The Postsynaptic Cell | GABRA3, GABRA2, GABRA4, GABRB1, GABRB2, GABRG2, GABRG3, CHRNA1, CHRNB4, CHRNA7, CHRND, CHRNE, GRIN2A, GRIN2B, CAMK2G, ADCY1, GRIA3, GRIA4, GRIK2, GRIK3, NCALD, DLG3, CAMK4, GNAL, ADCY2, ADCY6, KCNJ10, GABBR1, GNGT1, GNG2, GNG4, GNG7, KCNJ12, KCNJ3, KCNJ6, KCNJ5, PLCB1, MAPK1, RAF1, BRAF, RASGRF2, RPS6KA2, PRKACB, GRIP1, AP2A1, PRKCA, PRKCB, EPB41L1, MYO6, MDM2, CACNG8 | 51 | 31.9 | 1.60 | 0.0002 | 0.0147 |
| 2424491 | DAP12 signaling | RAF1, VAV2, GRAP2, PLCG2, SHC1, SOS1, ADCY2, ADCY6, ADCY1, AKT2, AKT3, FOXO1, FOXO4, GSK3B, MDM2, TSC2, PDE1C, CAMK4, PRKACB, PRKCA, PRKAR2A, ITPR3, ITPR1, ITPR2, MAPK1, TNRC6A, TNRC6B, AGO2, AGO1, AGO3, THEM4, PTEN, PHLPP2, PIK3CA, PIK3CB, KLRK1, TRAT1, KIT, IRS1, IRS2, GAB1, FRS2, CD86, CD28, EGFR, FGFR1, KLB, ERBB3, ERBB4, FGF1, FGF2, FGF10, NRG2, NRG1, EREG, NRG4, RICTOR | 57 | 35.8 | 1.59 | 7.32807E-05 | 0.0147 |
| 1227986 | Signaling by ERBB2 | RAF1, ADCY2, ADCY6, ADCY1, AKT2, AKT3, FOXO1, FOXO4, GSK3B, MDM2, TSC2, GAB1, EGFR, ERBB4, EREG, NRG1, NRG4, NRG2, ERBB3, PDE1C, CAMK4, PRKACB, PRKCA, PRKAR2A, ERBB2IP, CUL5, ITPR3, ITPR1, ITPR2, RNF41, MAPK1, SOS1, TNRC6A, TNRC6B, AGO2, AGO1, AGO3, THEM4, PTEN, PHLPP2, PIK3CA, RICTOR, TRAT1, KIT, IRS1, IRS2, FRS2, CD86, CD28, PIK3CB, FGFR1, KLB, FGF1, FGF2, FGF10, SHC1 | 56 | 35.4 | 1.58 | 9.83039E-05 | 0.0147 |
| 1236394 | Signaling by ERBB4 | RAF1, AKT2, AKT3, FOXO1, FOXO4, GSK3B, MDM2, TSC2, ERBB4, EREG, NRG1, NRG4, NRG2, APH1B, APH1A, PIK3CA, MAPK1, EGFR, ERBB3, NCOR1, ESR1, PRLR, TNRC6A, TNRC6B, AGO2, AGO1, AGO3, THEM4, PTEN, PHLPP2, WWOX, PGR, BTRC, SKP1, RBX1, SHC1, RICTOR, TRAT1, KIT, IRS1, IRS2, GAB1, FRS2, CD86, CD28, PIK3CB, FGFR1, KLB, FGF1, FGF2, FGF10, SOS1 | 52 | 33.1 | 1.57 | 0.0002 | 0.0147 |
| 186763 | Downstream signal transduction | RAF1, ADCY2, ADCY6, ADCY1, AKT2, AKT3, FOXO1, FOXO4, GSK3B, MDM2, TSC2, PDE1C, CAMK4, PRKACB, PRKCA, PRKAR2A, ITPR3, ITPR1, ITPR2, MAPK1, SOS1, TNRC6A, TNRC6B, AGO2, AGO1, AGO3, THEM4, PTEN, PHLPP2, BCAR1, PIK3CA, PIK3CB, TRAT1, KIT, IRS1, IRS2, GAB1, FRS2, CD86, CD28, EGFR, FGFR1, KLB, ERBB3, ERBB4, FGF1, FGF2, FGF10, NRG2, NRG1, EREG, NRG4, RICTOR, STAT1, STAT6 | 55 | 35.4 | 1.56 | 0.0002 | 0.0147 |
| 2454202 | Fc epsilon receptor (FCERI) signaling | MAPK10, RAF1, PPP3R1, PPP3CB, VAV2, GRAP2, PLCG2, SHC1, SOS1, TAB1, TAB3, AKT2, AKT3, FOXO1, FOXO4, GSK3B, MDM2, TSC2, MAP3K1, ITK, TXK, PAK2, MAPK1, NFATC1, NFATC3, NFATC2, ITPR3, ITPR1, ITPR2, MAP2K4, TNRC6A, TNRC6B, AGO2, AGO1, AGO3, THEM4, PTEN, PHLPP2, GAB2, PIK3CA, PIK3CB, TRAT1, KIT, IRS1, IRS2, GAB1, FRS2, CD86, CD28, EGFR, FGFR1, KLB, ERBB3, ERBB4, FGF1, FGF2, FGF10, NRG2, NRG1, EREG, NRG4, RICTOR | 62 | 39.9 | 1.55 | 8.44294E-05 | 0.0147 |
| 177929 | Signaling by EGFR | RAF1, GAB1, EGFR, ADCY2, ADCY6, ADCY1, AKT2, AKT3, FOXO1, FOXO4, GSK3B, MDM2, TSC2, EPS15L1, CBL, AP2A1, STAM2, CDC42, PDE1C, CAMK4, PRKACB, PRKCA, PRKAR2A, ITPR3, ITPR1, ITPR2, MAPK1, PIK3CA, PTEN, RICTOR, THEM4, TRAT1, TNRC6A, TNRC6B, AGO2, KIT, IRS1, IRS2, PHLPP2, AGO1, AGO3, FRS2, CD86, CD28, PIK3CB, FGFR1, KLB, ERBB3, ERBB4, FGF1, FGF2, FGF10, NRG2, NRG1, EREG, NRG4, SOS1, SHC1, ADAM12 | 59 | 38.6 | 1.53 | 0.0002 | 0.0147 |
| 166520 | Signalling by NGF | BRAF, KIDINS220, FRS2, RAF1, ADCYAP1, NTRK2, IRS1, IRS2, PIK3CA, PIK3CB, ADCY2, ADCY6, ADCY1, AKT2, AKT3, FOXO1, FOXO4, GSK3B, MDM2, TSC2, MAPK1, DNAL4, AP2A1, SORCS3, PDE1C, CAMK4, PRKACB, PRKCA, PRKAR2A, MAGED1, BCL2L11, SQSTM1, RASGRF2, VAV2, PLEKHG2, MCF2L, ARHGEF11, SOS1, ABR, AKAP13, ARHGEF4, FGD2, ITSN1, KALRN, OBSCN, APH1B, APH1A, RPS6KA5, RPS6KA2, ITPR3, ITPR1, ITPR2, DNM3, ELK1, MEF2C, PPP2R5D, PPP2R1B, DUSP6, SHC1, TNRC6A, TNRC6B, AGO2, AGO1, AGO3, THEM4, PTEN, PHLPP2, PCSK6, PCSK5, RIT1, RICTOR, TRAT1, KIT, GAB1, CD86, CD28, EGFR, FGFR1, KLB, ERBB3, ERBB4, FGF1, FGF2, FGF10, NRG2, NRG1, EREG, NRG4, PRKCI, HDAC2 | 90 | 64.0 | 1.41 | 0.0002 | 0.0147 |
| 180292 | GAB1 signalosome | GAB1, EGFR, AKT2, AKT3, FOXO1, FOXO4, GSK3B, MDM2, TSC2, TNRC6A, TNRC6B, AGO2, AGO1, AGO3, THEM4, PTEN, PHLPP2, PIK3CA, TRAT1, KIT, IRS1, IRS2, FRS2, CD86, CD28, PIK3CB, FGFR1, KLB, ERBB3, ERBB4, FGF1, FGF2, FGF10, NRG2, NRG1, EREG, NRG4, RICTOR | 38 | 23.0 | 1.66 | 0.0005 | 0.0262 |
| 1280218 | Adaptive Immune System | REL, CDC42, CD86, CD28, RAPGEF3, HLA-DPA1, HLA-DQA2, HLA-DQA1, HLA-DPB1, HLA-DQB2, PRKCB, SKP1, BTRC, PSMA2, PSMA6, PSMD11, PSMD7, PSMD9, PAK2, RAP1B, RASGRP3, AKT2, AKT3, FOXO1, FOXO4, GSK3B, MDM2, TSC2, NCF4, DAPP1, SOS1, CBL, PLCG2, SHC1, ITPR3, ITPR1, ITPR2, PDIA3, SEC31A, CANX, HSPA5, TAP2, SEC24B, SEC24C, HLA-B, LNPEP, SEC61A2, UBA6, ATG7, UBE2D2, UBE2D4, UBE2E1, UBE2E3, UBE2L6, UBE2W, DTX3L, HERC2, MGRN1, NEDD4L, RNF144B, TRIM32, TRIM37, UBR1, UBR2, GAN, RCHY1, UBE3B, UBE3C, UBR4, RNF41, SMURF2, FBXO27, FBXO44, RNF4, ARIH2, RNF111, WSB1, RBX1, CUL2, CUL3, FBXW8, CDC27, BLMH, SKP2, FBXO6, TCEB1, KLHL21, KLHL9, ZBTB16, CUL5, ASB1, ASB8, SOCS3, BTLA, PIK3CA, GRAP2, RICTOR, CD74, CTSA, CTSB, CTSC, CTSE, CTSK, CTSO, ICOSLG, CD274, PPP2R5D, PPP2R5C, PPP2R5E, PPP2R1B, PVRL2, CXADR, HLA-DOB, PTEN, THEM4, TRAT1, TNRC6A, TNRC6B, AGO2, KIT, IRS1, IRS2, GAB1, PHLPP2, AGO1, AGO3, FRS2, PIK3CB, EGFR, FGFR1, KLB, ERBB3, ERBB4, FGF1, FGF2, FGF10, NRG2, NRG1, EREG, NRG4, FCGR3A, ITK, ENAH, EVL, ITGB2, CD226, KIR3DL3, KIR2DL3, KIR2DL1, KIR2DS4, KIR3DL1, KIR2DL4, KIR2DL2, KIR2DS5, KIR2DS1, ULBP3, ULBP1, KLRK1, LGMN, DNM3, CLTC, AP2A1, RAB7A, DCTN5, DCTN3, DYNC1LI2, KIF3A, DYNC2H1, KIF5A, KIF23, TUBA1A, PRKACB, RAF1 | 173 | 139.8 | 1.24 | 0.0006 | 0.0320 |
| 1433557 | Signaling by SCF-KIT | RAF1, SOS1, KIT, AKT2, AKT3, FOXO1, FOXO4, GSK3B, MDM2, TSC2, MAPK1, STAT1, PIK3CA, GAB2, GRAP2, GRB10, FER, TNRC6A, TNRC6B, AGO2, AGO1, AGO3, THEM4, PTEN, PHLPP2, TRAT1, IRS1, IRS2, GAB1, FRS2, CD86, CD28, PIK3CB, EGFR, FGFR1, KLB, ERBB3, ERBB4, FGF1, FGF2, FGF10, NRG2, NRG1, EREG, NRG4, RICTOR, PRKCA, CBL | 48 | 31.2 | 1.54 | 0.0007 | 0.0341 |

Table G. Reactome pathways significantly (p-value <= 0.05) enriched among predicted target genes of each selected isomiRs belonging to miRNAs and moRNAs differentially expressed in PMF vs CTR samples.

| **Reactome Pathway** | | **isomiR** | **miRNA** | **Gene symbols** | **Obs. count** | **Exp. count** | **Fold enrich.** | **p-value** |
| --- | --- | --- | --- | --- | --- | --- | --- | --- |
| **ID** | **Name** |  |  |  |  |  |  |  |
| 112314 | Neurotransmitter Receptor Binding And Downstream Transmission In The Postsynaptic Cell | hsa-3p-moR-128-2-LONGER | hsa-3p-moR-128-2 | GABRG2, CHRNB4, CHRNA7, GRIN2A, GRIN2B, CAMK2G, GRIK3, ADCY2, GABBR1, GNG7, MAPK1, BRAF, RASGRF2, PRKCA, CACNG8 | 15 | 5.41 | 2.77 | 0.0003 |
| 1226099 | Signaling by FGFR in disease | hsa-3p-moR-128-2 | hsa-3p-moR-128-2 | MAPK1, FGF10, SHC1, MDM2, GAB2, PDE1C, PRKACB, PRKCA, FGFR1OP, TNRC6A, TNRC6B, AGO1 | 12 | 5.42 | 2.21 | 0.0079 |
|  |  | hsa-miR-10b-5p-FC_T | hsa-miR-10b-5p | GSK3B, MDM2, GAB2, PRKAR2A, ITPR2, TNRC6B, IRS1, AGO3, CD28, NRG2, MKNK1 | 11 | 4.81 | 2.29 | 0.0086 |
| 1227986 | Signaling by ERBB2 | hsa-miR-29a-3p-EXACT_HIGHER | hsa-miR-29a-3p | AKT2, AKT3, EREG, NRG1, CAMK4, ITPR3, TNRC6A, AGO1, AGO3, IRS1 | 10 | 4.04 | 2.47 | 0.0071 |
| 157118 | Signaling by NOTCH | hsa-miR-10b-5p-FC_T | hsa-miR-10b-5p | MAMLD1, TNRC6B, AGO3, NCOR1, NCOR2, HDAC11, HDAC4, HDAC8, RBX1 | 9 | 2.85 | 3.15 | 0.0021 |
| 177929 | Signaling by EGFR | hsa-miR-29a-3p-EXACT_HIGHER | hsa-miR-29a-3p | AKT2, AKT3, CDC42, CAMK4, ITPR3, TNRC6A, IRS1, AGO1, AGO3, NRG1, EREG, ADAM12 | 12 | 4.41 | 2.72 | 0.0015 |
| 186763 | Downstream signal transduction | hsa-miR-29a-3p-EXACT_HIGHER | hsa-miR-29a-3p | AKT2, AKT3, CAMK4, ITPR3, TNRC6A, AGO1, AGO3, IRS1, NRG1, EREG | 10 | 4.04 | 2.47 | 0.0071 |
| 190236 | Signaling by FGFR | hsa-miR-29a-3p-EXACT_HIGHER | hsa-miR-29a-3p | AKT2, AKT3, CAMK4, ITPR3, TNRC6A, IRS1, AGO1, AGO3, NRG1, EREG | 10 | 3.99 | 2.51 | 0.0065 |
| 190333 | Downstream signaling of activated FGFR | hsa-miR-29a-3p-EXACT_HIGHER | hsa-miR-29a-3p | AKT2, AKT3, CAMK4, ITPR3, TNRC6A, AGO1, AGO3, IRS1, NRG1, EREG | 10 | 3.73 | 2.68 | 0.0040 |
| 1912408 | Pre-NOTCH Transcription and Translation | hsa-miR-19b-3p-HIGHER | hsa-miR-19b-3p | CCND1, TFDP2, TNRC6B | 3 | 0.40 | 7.53 | 0.0068 |
|  |  | hsa-miR-29a-3p-EXACT_HIGHER | hsa-miR-29a-3p | TNRC6A, AGO1, AGO3, NOTCH2 | 4 | 0.50 | 8.02 | 0.0013 |
| 1912422 | Pre-NOTCH Expression and Processing | hsa-miR-29a-3p-EXACT_HIGHER | hsa-miR-29a-3p | NOTCH2, TNRC6A, AGO1, AGO3 | 4 | 0.76 | 5.25 | 0.0065 |
| 2172127 | DAP12 interactions | hsa-miR-379-5p-EXACT_HIGHER | hsa-miR-379-5p | TSC2, PDE1C, RICTOR, TNRC6A, KIT, AGO1, CD86, FGF1, FGF2, KIR2DL2, KIR2DS5, KIR2DS1, KIR3DL3, KIR2DL3, KIR2DL1, KIR2DS4, CLEC5A | 17 | 7.88 | 2.16 | 0.0022 |
|  |  | hsa-miR-29a-3p-EXACT_HIGHER | hsa-miR-29a-3p | GRAP2, AKT2, AKT3, CAMK4, ITPR3, TNRC6A, IRS1, AGO1, AGO3, NRG1, EREG | 11 | 4.46 | 2.46 | 0.0050 |
| 2424491 | DAP12 signaling | hsa-miR-29a-3p-EXACT_HIGHER | hsa-miR-29a-3p | GRAP2, AKT2, AKT3, CAMK4, ITPR3, TNRC6A, AGO1, AGO3, IRS1, NRG1, EREG | 11 | 4.10 | 2.69 | 0.0026 |
| 2454202 | Fc epsilon receptor (FCERI) signaling | hsa-miR-29a-3p-EXACT_HIGHER | hsa-miR-29a-3p | MAPK10, GRAP2, AKT2, AKT3, ITPR3, TNRC6A, AGO1, AGO3, IRS1, NRG1, EREG | 11 | 4.57 | 2.41 | 0.0059 |
| 2559585 | Oncogene Induced Senescence | hsa-3p-moR-128-2 | hsa-3p-moR-128-2 | MAPK1, MDM2, TNRC6A, TNRC6B, AGO1 | 5 | 0.91 | 5.50 | 0.0018 |
|  |  | hsa-miR-10b-5p-FC_T | hsa-miR-10b-5p | CDK6, ETS1, MDM2, TNRC6B, AGO3 | 5 | 0.81 | 6.19 | 0.0011 |
|  |  | hsa-miR-10b-5p-HIGHER | hsa-miR-10b-5p | CDK6, ETS1, MDM2 | 3 | 0.37 | 8.16 | 0.0057 |
| 426496 | Post-transcriptional silencing by small RNAs | hsa-3p-moR-128-2 | hsa-3p-moR-128-2 | TNRC6A, TNRC6B, AGO1 | 3 | 0.23 | 13.21 | 0.0011 |
|  |  | hsa-miR-29a-3p-EXACT_HIGHER | hsa-miR-29a-3p | TNRC6A, AGO1, AGO3 | 3 | 0.18 | 16.32 | 0.0006 |
| 429914 | Deadenylation-dependent mRNA decay | hsa-miR-10b-5p-FC_T | hsa-miR-10b-5p | LSM1, PATL1, XRN1, CNOT6, EXOSC9 | 5 | 1.33 | 3.77 | 0.0099 |
|  |  | hsa-miR-19b-3p-HIGHER | hsa-miR-19b-3p | PATL1, EIF4G1, CNOT4, CNOT6, CNOT8 | 5 | 0.96 | 5.18 | 0.0026 |

**Table H:** Reactome pathways Significantly (p-value <= 0.05) enriched in targets of 3-moR-128-2.

| **Reactome ID** | **Pathway** | **P-value** | **Genes** |
| --- | --- | --- | --- |
| 112314 | Neurotransmitter Receptor Binding And Downstream Transmission In The Postsynaptic Cell | 0.0002 | GABRG2, CHRNA1, CHRNB4, CHRNA7, GRIN2A, GRIN2B, CAMK2G, GRIK3, GNAL, ADCY2, GABBR1, GNG7, MAPK1, BRAF, RASGRF2, PRKACB, PRKCA, MYO6, MDM2, CACNG8 |
| 426496 | Post-transcriptional silencing by small RNAs | 0.0004 | TNRC6A, TNRC6B, AGO1, AGO3 |
| 2559585 | Oncogene Induced Senescence | 0.0009 | MAPK1, CDK6, MDM2, TNRC6A, TNRC6B, AGO1, AGO3 |
| 189451 | Heme biosynthesis | 0.0028 | ALAD, COX15, CPOX, FECH |
| 211000 | Regulatory RNA pathways | 0.0033 | AGO1, AGO3, RAN, POLR2H, TNRC6A, TNRC6B |
| 4086398 | Ca2+ pathway | 0.0034 | PPP3R1, NLK, NFATC1, FZD3, GNG7, TNRC6A, TNRC6B, AGO1, AGO3, PRKCA |
| 168274 | Export of Viral Ribonucleoproteins from Nucleus | 0.0034 | XPO1, RAN |
| 168333 | NEP/NS2 Interacts with the Cellular Export Machinery | 0.0034 | XPO1, RAN |
| 1226099 | Signaling by FGFR in disease | 0.0040 | KLB, MAPK1, FGF10, SHC1, ADCY2, MDM2, BRAF, GAB2, GAB1, PDE1C, PRKACB, PRKCA, FGFR1OP, TRIM24, TNRC6A, TNRC6B, AGO1, AGO3, ERBB3 |
| 112315 | Transmission across Chemical Synapses | 0.0041 | GABRG2, CHRNA1, CHRNB4, CHRNA7, GRIN2A, GRIN2B, CAMK2G, GRIK3, GNAL, ADCY2, GABBR1, GNG7, MAPK1, BRAF, RASGRF2, PRKACB, ALDH5A1, PRKCA, MYO6, MDM2, CACNG8 |
| 438064 | Post NMDA receptor activation events | 0.0043 | CAMK2G, MAPK1, RASGRF2, GRIN2A, GRIN2B, PRKACB, BRAF |
| 442742 | CREB phosphorylation through the activation of Ras | 0.0049 | MAPK1, RASGRF2, GRIN2A, GRIN2B, CAMK2G, BRAF |
| 157118 | Signaling by NOTCH | 0.0050 | JAG2, DTX4, MIB1, APH1B, TLE4, TNRC6A, TNRC6B, AGO1, AGO3, HDAC8, SKP1, RBX1, ST3GAL3 |
| 442755 | Activation of NMDA receptor upon glutamate binding and postsynaptic events | 0.0079 | GRIN2A, GRIN2B, CAMK2G, MAPK1, RASGRF2, PRKACB, BRAF |
| 418990 | Adherens junctions interactions | 0.0097 | CDH18, CDH6, CDH8, MLLT4, CADM1, CADM2 |
| 1236394 | Signaling by ERBB4 | 0.0100 | MDM2, APH1B, MAPK1, ERBB3, ESR1, TNRC6A, TNRC6B, AGO1, AGO3, PGR, SKP1, RBX1, SHC1, GAB1, KLB, FGF10 |
